# Unique Cell Adhesion and Invasion Properties of *Yersinia enterocolitica* O:3, the Most Frequent Cause of Human Yersiniosis

**DOI:** 10.1371/journal.ppat.1002117

**Published:** 2011-07-07

**Authors:** Frank Uliczka, Fabio Pisano, Julia Schaake, Tatjana Stolz, Manfred Rohde, Angelika Fruth, Eckhard Strauch, Mikael Skurnik, Julia Batzilla, Alexander Rakin, Jürgen Heesemann, Petra Dersch

**Affiliations:** 1 Department of Molecular Infection Biology, Helmholtz-Zentrum für Infektionsforschung, Braunschweig, Germany; 2 Institut für Mikrobiologie, Technische Universität Braunschweig, Braunschweig, Germany; 3 Department of Medical Microbiology, Helmholtz-Zentrum für Infektionsforschung, Braunschweig, Germany; 4 Robert Koch-Institut, Wernigerode, Germany; 5 Bundesinstitut für Risikoforschung, Berlin, Germany; 6 Department of Bacteriology and Immunology, The Haartman Institute, University of Helsinki and Helsinki University Central Hospital Laboratory Diagnostics, Helsinki, Finland; 7 Max von Pettenkofer Institut, Ludwigs-Maximilians-Universität, München, Germany; Yale University School of Medicine, United States of America

## Abstract

Many enteric pathogens are equipped with multiple cell adhesion factors which are important for host tissue colonization and virulence. *Y. enterocolitica*, a common food-borne pathogen with invasive properties, uses the surface proteins invasin and YadA for host cell binding and entry. In this study, we demonstrate unique cell adhesion and invasion properties of *Y. enterocolitica* serotype O:3 strains, the most frequent cause of human yersiniosis, and show that these differences are mainly attributable to variations affecting the function and expression of invasin in response to temperature. In contrast to other enteric *Yersinia* strains, invasin production in O:3 strains is constitutive and largely enhanced compared to other *Y. enterocolitica* serotypes, in which *invA* expression is temperature-regulated and significantly reduced at 37°C. Increase of invasin levels is caused by (i) an IS1667 insertion into the *invA* promoter region, which includes an additional promoter and RovA and H-NS binding sites, and (ii) a P98S substitution in the *invA* activator protein RovA rendering the regulator less susceptible to proteolysis. Both variations were shown to influence bacterial colonization in a murine infection model. Furthermore, we found that co-expression of YadA and down-regulation of the O-antigen at 37°C is required to allow efficient internalization by the InvA protein. We conclude that even small variations in the expression of virulence factors can provoke a major difference in the virulence properties of closely related pathogens which may confer better survival or a higher pathogenic potential in a certain host or host environment.

## Author Summary

Bacterial infections are generally initiated by molecular interactions that occur between the pathogen and its host cell. These interactions are usually mediated by adhesion and invasion factors exposed from the surface of the bacteria which are necessary for the colonization of host tissues and fundamental to pathogenesis. It is well known that many bacterial species contain several different adhesin determinants, which often vary between bacteria of the same species, reflecting the fact that each microbe has adapted to a distinct ecological niche. Here, we show that also small alterations changing the expression pattern of adhesins and virulence gene regulators in response to environmental factors (e.g. temperature) lead to fundamental differences in pathogen-host cell interactions and pathogenesis. Modulation of virulence gene expression constitutes an ideal mechanism to adjust virulence-associated processes of pathogens to different hosts (e.g. with varying body temperature) as it allows the bacteria to readjust expression of certain gene subsets of regulatory networks controlling virulence, stress and metabolic adaptation to their demands in individual hosts.

## Introduction

*Yersinia enterocolitica* is a common gram-negative zoonotic pathogen that is able to grow in the environment and cause enteric diseases (Yersiniosis), ranging from enteritis, severe diarrhea, mesenteric lymphadenitis, hepatic or splenic abscesses to postinfectious extraintestinal sequelae such as reactive arthritis and erythema nodosum [1]. Infection by *Y. enterocolitica* is usually initiated through uptake of contaminated food or water. Following ingestion, the bacteria first colonize the lumen and transmigrate through antigen-sampling M cells across the epithelial lining of the small intestine, resulting in the colonization of the underlying lymphoid tissues (Peyer's patches) [2], [3]. Subsequently, *Y. enterocolitica* can spread via the lymph and/or blood into the mesenteric lymph nodes or to extraintestinal sites such as liver and spleen [4], [5], [6]. Alternatively, the bacteria may bypass colonization of the Peyer's patches and spread directly from the intestine to the systemic tissues, similar to what has been observed for enteropathogenic *Yersinia pseudotuberculosis* [7], [8].

Adhesion, invasion and survival in deeper tissues depend on several *Yersinia* virulence factors encoded on the *Yersinia* chromosome or the 65–70 kb virulence plasmid (pYV) [9]. *Y. enterocolitica* produces at least three invasion factors, invasin, Ail (attachment-invasion locus), and YadA (*Yersinia* adhesin A) which were shown to promote adherence to and invasion into mammalian cells [10], [11], [12]. Invasin, the primary invasion factor, binds with high affinity to beta 1 chain integrin receptors found on the surface of M cells but not on the apical side of brush border cells, and mediates efficient and rapid internalization into host cells [13], [14]. As invasin is strongly expressed at environmental temperature but only weakly at 37°C, it is assumed to support initial colonization and survival of host tissues during the very early stages of an infection [15], [16], [17]. Recent studies showed that the dimeric winged-helix transcriptional regulator RovA controls transcription of the invasin gene (*invA*) in response to temperature. For this purpose, RovA uses an in-built thermosensor to control its DNA-binding activity and its susceptibility to the proteolytic degradation by ATP-dependent proteases [18].

After the initiation of the infection, the YadA and Ail proteins seem to be the predominant adhesins in infected tissues. Both virulence factors mediate serum resistance and promote tight adherence to extracellular matrix proteins, such as fibronectin and/or collagen, but their contribution to bacterial uptake is relatively small [19], [20], [21], [22], [23], [24], [25]. The *yadA* gene is located on pYV and its expression, together with the plasmid-encoded type III secretion system (Ysc proteins) and the antiphagocytic effector proteins (Yops) is controlled by the VirF(LcrF) activator. VirF-dependent induction of *yadA*, *yop* and *ysc* expression occurs exclusively at 37°C [26], [27]. Ail is also predominantly expressed at 37°C, and regulated by pH and oxygen content, but the control mechanisms are still unclear [24].

Besides the classical pathogenicity factors, other surface factors also contribute or are required for full virulence. Lipopolysaccharides (LPS) of *Y. enterocolitica* serotypes O:3 and O:8 are required for successful colonization of the gut and play an important role in the outer membrane integrity of the bacteria [28], [29], [30]. LPS O polysaccharide (O-antigen) mutants were attenuated in virulence and impaired in their ability to colonize the Peyer's patches, liver and spleen [30], [31]. Production of the O-antigen is also temperature-regulated with maximal expression at moderate temperatures [32], [33]. A complex network regulates O-antigen expression at the transcriptional level and the RosA/RosB efflux pump/potassium antiporter system and Wzz, the O-antigen chain length determinant, are indirectly involved in the temperature-dependent control process [33]. In addition, flagella-dependent motility is required to initiate host cell invasion by ensuring migration and cell contact of the bacteria [34].

Most studies on *Y. enterocolitica* virulence factors and their contribution to virulence were performed using highly mouse-virulent bioserogroup 1B/O:8 strains, in particular *Y. enterocolitica* 8081v. However, several other human pathogenic *Y. enterocolitica* strains which are less virulent in mice (e.g. serotypes O:3, O:9 and O:5,27) were also frequently isolated from patients [1]. Among these strains, *Y. enterocolitica* bioserotype 4/O:3 is by far the most frequent cause of human yersiniosis in Europe and Japan (80–90%). *Y. enterocolitica* infections are less common in North America. However, since the 1980s, serogroup O:3 strains have emerged as an occasional cause of foodborne outbreaks and replaced O:8 as the predominant serotype of *Y. enterocolitica* reported to CDC [35], [36], [37], [38]. They mainly originate from domestic pigs (prevalence of 0–65% in fattening pig herds), which are often asymptotic carriers, and in which they commonly colonize the lymphoid tissue of the gut and oropharynx [39], [40]. As only very little is known about the pathogenicity of *Y. enterocolitica* bioserotype 4/O:3, we compared host cell interactions of different human-, pig- and food-derived *Y. enterocolitica* isolates and found that expression and function of surface-exposed virulence factors of serotype O:3 strains differ significantly from other *Y. enterocolitica* serotypes. This may reflect an adaptation of *Y. enterocolitica* O:3 to the intestine of pigs which make them also highly pathogenic for humans.

## Results

### *Y. enterocolitica* O:3 interaction with epithelial cells differs significantly from other *Y. enterocolitica* serotypes

**Figure 1:**
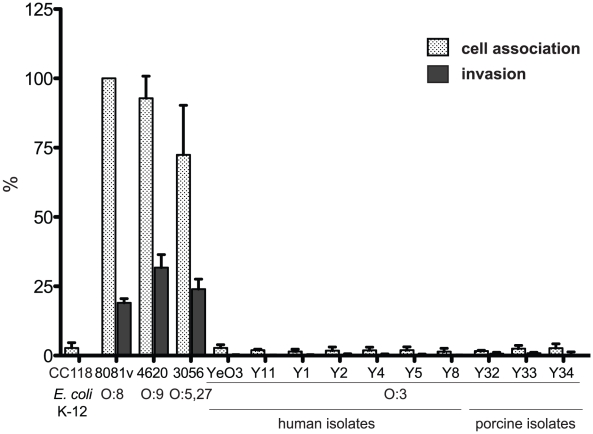
*Y. enterocolitica* O:3 interaction with epithelial cells. Ten different *Y. enterocolitica* serotype O:3 isolates from human patients or pigs, *Y. enterocolitica* O:8 strain 8081v, *Y. enterocolitica* O:9 strain 4620 and *Y. enterocolitica* O:5,27 strain 3056 were grown at 25°C overnight in LB medium. About 5·10^4^ HEp-2 cells were infected with 5·10^5^ bacteria and incubated at 22–25°C to monitor cell association or 37°C to determine the internalization efficiency of the bacteria by the gentamicin protection assay. *E. coli* K-12 was used as negative control. Data are presented as means ± standard deviations of three independent experiments performed in duplicate.

In order to obtain information about interactions of *Y. enterocolitica* serotype O:3 (YeO:3) strains with host cells, we first investigated the adhesion and invasion efficiency of two reference strains Y11 and YeO3 and 25 different YeO:3 strains isolated from human patients, animals or food between 2005 and 2008 in Germany (Table **S1**). None of the serotype O:3 isolates was able to efficiently bind and invade into cultured human epithelial cells when the bacteria were grown at standard culture conditions and similar patterns of host-cell associated bacteria (adhesion and invasion) were obtained when infection was performed at 22–25°C or 37°C (Fig. **1**, **S1**, data not shown). A prolongation of the infection time from 30 min to 3 hours and/or use of other human, porcine and murine epithelial cell lines did not significantly enhance the efficiency of cell adherence (data not shown), indicating that low-efficiency of adhesion and invasion is independent of the cell line and host species. In contrast, all other tested *Y. enterocolitica* isolates (serotypes O:5,27, O:8 and O:9) adhered very efficiently and were able to enter all tested epithelial cell lines after 30 min with a frequency ranging from 20–30% depending on the serotype and the isolate (Fig. **1**, data not shown).

### Amotility of *Y. enterocolitica* O:3 affects cell invasion efficiency

**Figure 2:**
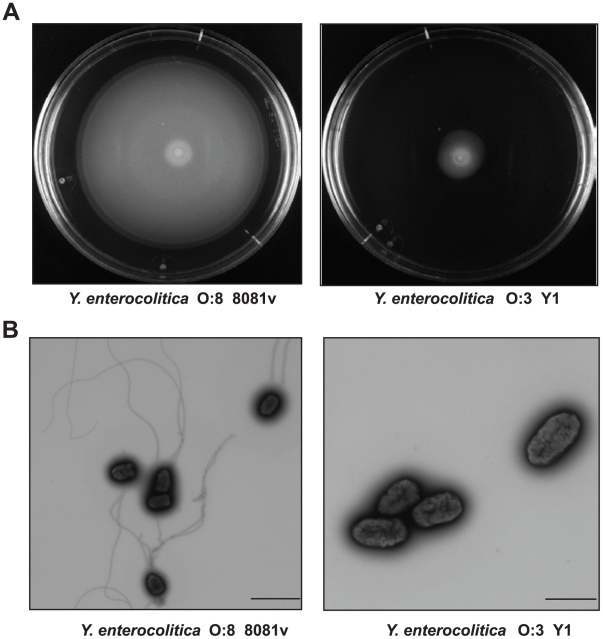
Motility and flagellation of *Y. enterocolitica* O:3 and O:8. (**A**) Swimming of *Y. enterocolitica* O:3 (Y1) and O:8 (8081v). Aliquots of 2 µl of the bacterial culture were inoculated onto LB swimming plates. The plates were incubated at 25°C for 48 h. (**B**) Transmission electron microscopy of *Y. enterocolitica* O:3 (Y1) and O:8 (8081v) grown to stationary phase. Bars indicate 2 µm and 1 µm, respectively.

It is known that motility is an important factor enhancing the invasion efficiency of yersiniae [34]. We tested motility of the YeO:3 strains and found that none of the isolates was motile on swimming and swarming agar plates in contrast to other *Y. enterocolitica* serotypes, e.g. *Y. enterocolitica* YeO:8 8081v (Fig. **2A**, data not shown). Transmission electron microscopy further revealed that YeO:3 strains are not flagellated (Fig. **2B**, data not shown), indicating that flagella synthesis is abolished or does not occur under used growth conditions (LB, 25°C). This phenotype was also observed with YeO:3 strains isolated from liver and spleen of BALB/c mice three days post infection (data not shown). Notably, 30–40% of the bacteria isolated from the intestine were flagellated (Fig. **S2**) and motile after *in vitro* cultivation for 24 h (data not shown). However, none of them remained motile and flagellated after 48 h, indicating that the bacteria are motile within the intestinal tract and rapidly repress flagella synthesis when grown on agar plates.

**Figure 3:**
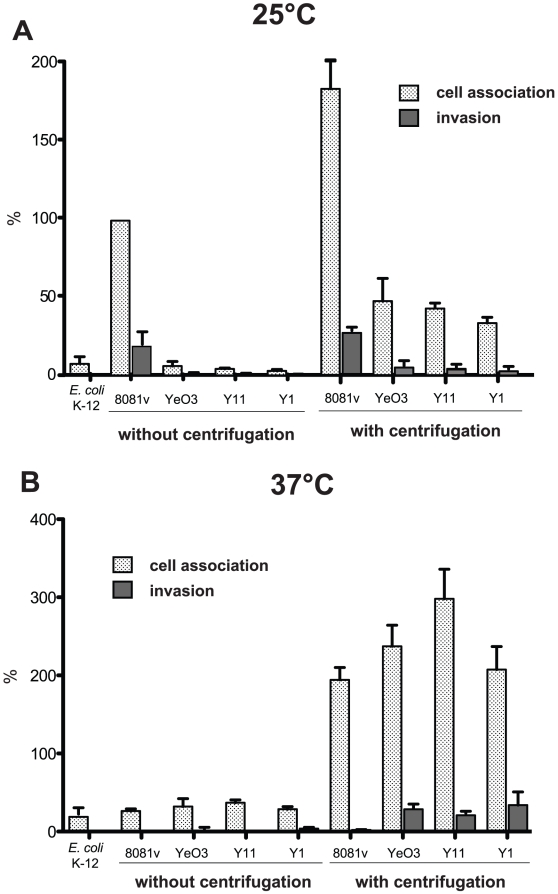
Host cell interaction of *Y. enterocolitica* O:3 by InvA at 25°C is less efficient due to amotility. Amotile *Y. enterocolitica* O:3 strains YeO3, Y11, and Y1 and motile *Y. enterocolitica* strain O:8 8081v were grown at 25°C (**A**) or 37°C (**B**) overnight. About 5·10^4^ HEp-2 cells were infected with 5·10^5^ bacteria and incubated with or without centrifugation of the bacteria onto the monolayer to monitor cell association (adhesion+invasion) or the internalization efficiency of the bacteria by the gentamicin protection assay. *E. coli* K-12 was used as negative control. Data are presented as means ± standard deviations of three independent experiments performed in duplicate.

It has been assumed that motility enhances the frequency of bacteria-cell interaction and/or provides an additional force for active cell entry. To investigate whether non-invasiveness of the serotype O:3 strains was caused by a reduction of host cell contacts due to amotility, we performed adhesion and invasion assays with or without centrifugation of the bacteria onto host cells (Fig. **3A**). When we pre-grew the bacteria at 25°C, adhesion and internalization was slightly increased after centrifugation, but the overall efficiency was still significantly lower compared to YeO:8 8081v. This demonstrated that amotility of the bacteria reduced host cell contact and invasion of YeO:3 grown at 25°C. However, this does not fully explain the observed differences. In this context, we also analyzed host cell adhesion and invasion of bacteria grown at 37°C (Fig. **3B**). Without centrifugation, the number of adherent YeO:8 8081v was significantly reduced and no invasion of the bacteria was detectable at 37°C. In contrast, YeO:8 8081v adhered tightly to HEp-2 cells after bacteria were artificially brought into cell contact by centrifugation, but they were not internalized (Fig. **3B**). This is consistent with previous studies showing that synthesis of the flagella and the primary internalization factor invasin is repressed at 37°C in *Y. enterocolitica* 8081v, whereas production of the major adhesion factor YadA is induced at 37°C but not at moderate growth temperatures [15], [41]. As shown in Fig. **3B**, pre-growth at 37°C and artificially induced host cell contact led to a significant raise of cell adhesion of all tested O:3 strains. Notably, only under these conditions efficient host cell invasion of YeO:3 strains was as efficient as cell uptake obtained with YeO:8 8081v grown at 25°C (20–30% of adherent bacteria). Since efficient cell adhesion and internalization of YeO:3 strains was only achieved after artificial host cell contact, in all following experiments bacteria were centrifugated onto host cells.

### Internalization of *Y. enterocolitica* O:3 into human epithelial cells

**Figure 4:**
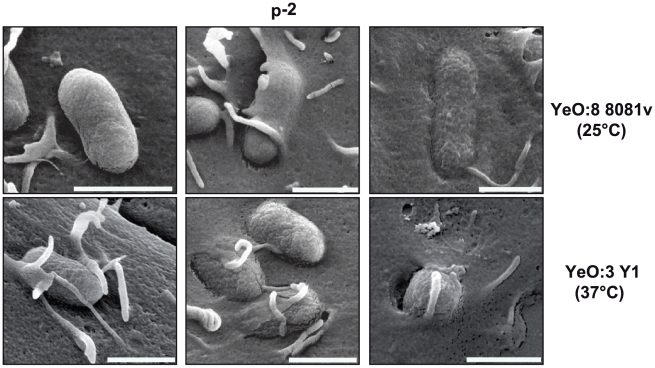
*Y. enterocolitica* O:3 and O:8 interaction with epithelial cells. *Y. enterocolitica* O:3 strain Y1 was pregrown at 37°C and *Y. enterocolitica* O:8 strain 8081v was grown at 25°C. The bacteria were added to HEp-2 and incubated for 30 min at 37°C after centrifugation of the bacteria onto the monolayer. Different stages of the internalization process are shown (initial binding, filopodia and lamellipodia formation). Bars indicate 1 µm.

Based on the previous experiments it seemed possible that an additional thermo-regulated internalization mechanism is responsible for host cell invasion at 37°C. To compare the invasion mechanism used by YeO:3 and YeO:8 strains, we monitored cell entry of YeO:8 8081v grown at 25°C and YeO:3 Y1 grown at 37°C into HEp-2 (Fig. **4A**) and Caco-2 (data not shown) cells by scanning electron microscopy. We found that adherence and invasion of both *Y. enterocolitica* serotypes showed very common features and were not cell type specific. After host cell binding, the cell surface in the vicinity of the microbes seems to be slightly drawn down, pseudopodia and lamellipodia are formed and the eukaryotic cell membrane then seems to enclose and surround the bacteria into a membrane-bound vacuole. In contrast, no cell adherence of YeO:3 strain Y1 was observed when grown at 25°C, and only simple attachment, but no formation of membrane protrusions was detectable when YeO:8 8081v was precultivated at 37°C (data not shown). This suggested that the internalization mechanism initiated by *Y. enterocolitica* serotype O:3 and O:8 strains is similar but expressed at different temperatures.

### Expression analysis of *Y. enterocolitica* O:3 invasin

**Figure 5:**
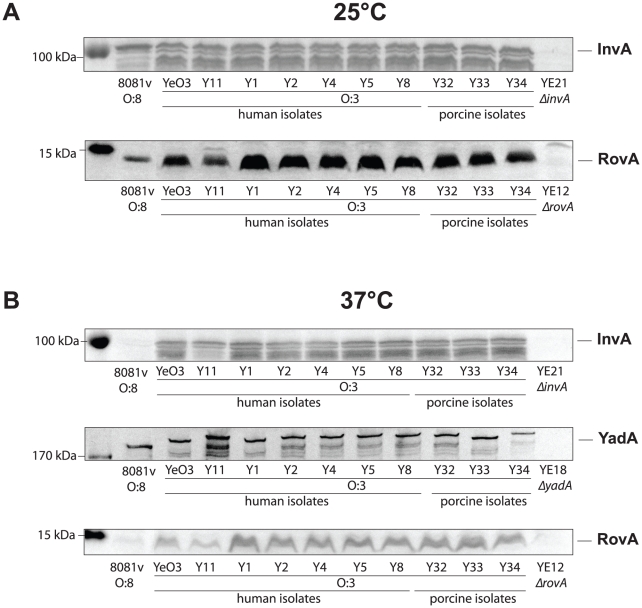
Expression analysis of *Y. enterocolitica* O:3 invasin, YadA, and RovA. *Y. enterocolitica* O:3 strains and the serotype O:8 reference strain 8081v were grown overnight at 25°C (**A**) and 37°C (**B**). Whole cell extracts for analysis of the DNA-binding protein RovA and the adhesins InvA and YadA were prepared, separated on SDS-polyacrylamid gels and analyzed by western blotting using polyclonal antibodies directed against RovA, InvA and YadA. A molecular marker the PageRuler Prestained Protein Ladder was loaded on the left.

In order to test this hypothesis, we analyzed the amount of invasin in both *Y. enterocolitica* serotypes and found that high amounts of the primary invasion factor invasin were present in all tested YeO:3 strains at 25°C and 37°C, whereas in YeO:8 8081v invasin was only detectable at 25°C, but not at 37°C (Fig. **5AB**). Production of invasin in YeO:3 strains at 37°C explains why the invasion rate is significantly enhanced at this growth temperature. However, this also raised the question why no internalization of the bacteria was observed when the bacteria were grown at 25°C, although similar amounts of the invasin protein were produced (Fig. **3**, **5**).

### *Y. enterocolitica* O:3 O-antigen blocks InvA-mediated invasion at 25°C

**Figure 6:**
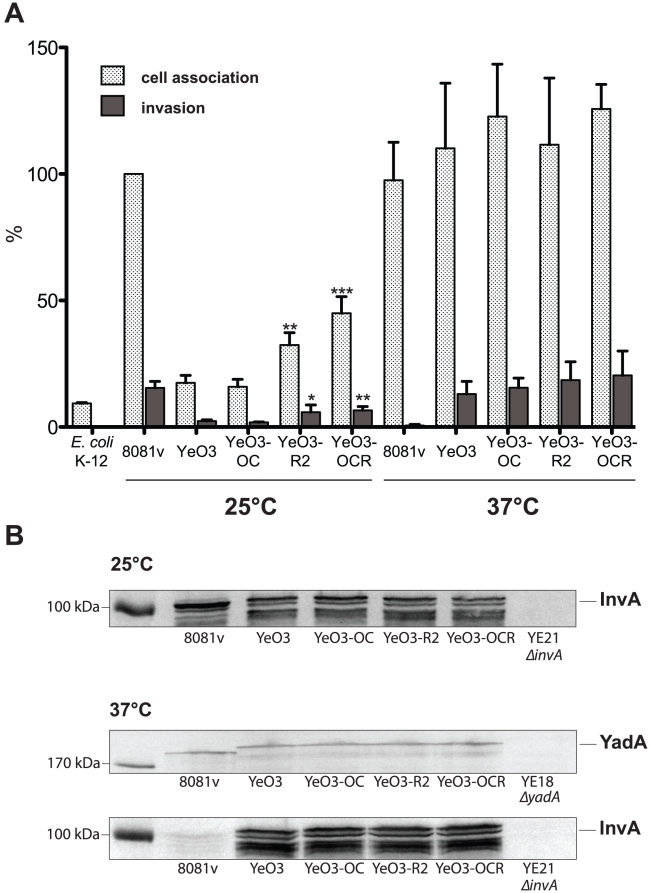
Influence of the *Y. enterocolitica* O:3 O-antigen on host cell invasion. *Y. enterocolitica* wildtype strains YeO3 and 8081v, and outer core and/or O-antigen deficient derivatives (YeO3-OC, YeO3-R2, YeO3-OCR) were grown at 25°C and 37°C. (**A**) About 5×10^4^ HEp-2 cells were infected with 5×10^5^ bacteria. After centrifugation of the bacteria onto the monolayer, cell association (adhesion+invasion) was monitored and internalization efficiency of the bacteria was determined by the gentamicin protection assay. *E. coli* K-12 was used as negative control. Data are presented as means ± standard deviations of three independent experiments performed in duplicate. Data were analyzed by the students t test. Stars indicate the results that differed significantly from those of YeO3 with * (P<0.05), ** (P<0.01), and *** (P<0.001) (**B**) Whole cell extracts were prepared from overnight cultures, separated on SDS-polyacrylamide gels and analyzed by western blotting using polyclonal antibodies directed against InvA and YadA. As a molecular marker the PageRuler Prestained Protein Ladder was loaded on the left.

To decipher the differences in the host cell invasion properties between the *Y. enterocolitica* O:3 and O:8 serotype, we first performed adhesion and invasion experiments with *E. coli* K-12 expressing the *invA*_O:3_ and *invA*_O:8_ genes and found a similar ability of both invasin proteins to promote cell attachment (25%) and entry (5%) (data not shown). This led to the hypothesis that a temperature-regulated surface structure might block invasin function of YeO:3 strains at moderate growth temperatures. Indeed, composition of the outer membrane and particularly make-up of LPS was shown to be strongly temperature-dependent in *Y. enterocolitica* [32]. Both the branched outer core hexasaccharide (OC) and the homopolymeric O-antigen (O-Ag) of the unique YeO:3 LPS are maximally produced below 30°C, whereas only very reduced levels of these LPS components are displayed on the bacterial surface at 37°C [32]. In order to investigate whether they sterically block the access of invasin to host cells, we used different mutant strains of *Y. enterocolitica* O:3 strain YeO3 deficient in O-Ag formation (YeO3-R2), OC biosynthesis (YeO3-OC), or both (YeO3-OCR) [42]. As shown in Fig. **6A**, both O-Ag deficient mutant strains (YeO3-R2, YeO3-OCR) have an increased capacity to interact and enter human epithelial cells, whereas no difference was detectable with the OC knock-out mutant (YeO3-OC). When the adhesion and uptake assays were performed at 37°C, the overall adhesion and invasion levels of the YeO3 wild-type strain were significantly increased and identical to that of YeO:8 8081v grown at 25°C, and no significant differences were observed in the absence of the O-Ag or the OC (Fig. **6A**). Notably, differences in host cell interactions did not result from differences in *invA* or *yadA* expression as identical amounts of invasin and YadA were detectable in the *Y. enterocolitica* O:3 wild-type YeO3 and the O-Ag and OC mutants grown at 25°C and 37°C (Fig. **6B**). Taken together, these data strongly suggest that the YeO:3 O-Ag reduces host cell interactions at 25°C, most likely through steric hindrance of adhesin/invasin host cell receptor binding. However, this does not seem to be the only reason why YeO3 is less invasive at 25°C than YeO8 8081v, as invasion of the O-Ag mutant strains was still lower compared to invasion of the *Y. enterocolitica* O:8 strain (Fig. **6A**).

### Co-expression of invasin and YadA is necessary for efficient invasion at 37°C

**Figure 7:**
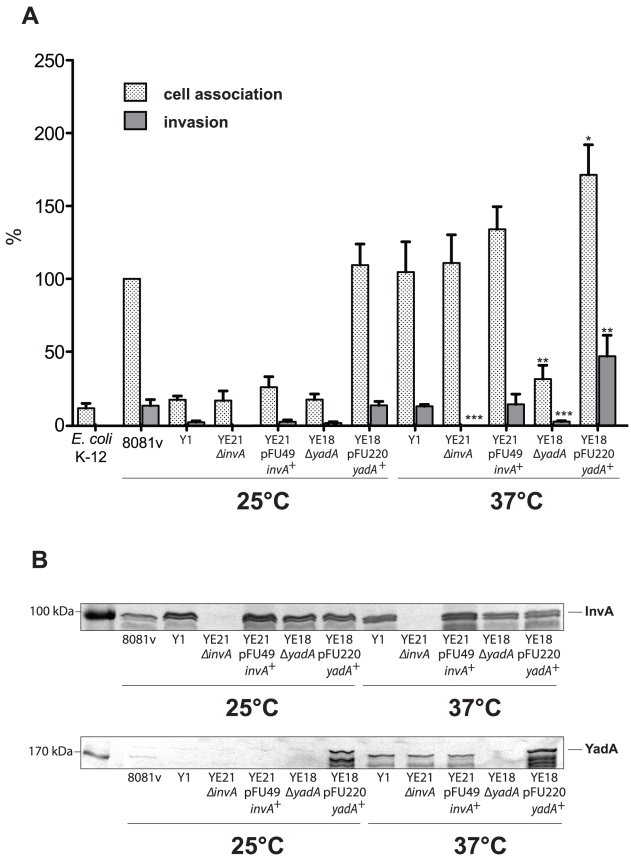
Coexpression of invasin and YadA is necessary for efficient invasion at 37°C. *Y. enterocolitica* strain O:8 strain 8081v, *Y. enterocolitica* O:3 strain Y1 and isogenic *invA* and *yadA* deficient mutant derivatives were grown overnight at 25°C and 37°C. (**A**) About 5·10^4^ HEp-2 cells were infected with 5·10^5^ bacteria and after centrifugation of the bacteria onto the monolayer the samples cell association (adhesion+invasion) was monitored and internalization efficiency of the bacteria was determined by the gentamicin protection assay. Data are presented as means ± standard deviations of three independent experiments performed in duplicate. Data were analyzed by the students t test. Data were analyzed by the students t test. Stars indicate the results that differed significantly from those of Y1 with * (P<0.05), ** (P<0.01), and *** (P<0.001). (**B**) Whole cell extracts were prepared from the overnight cultures, separated on SDS-polyacrylamide gels and analyzed by western blotting using polyclonal antibodies directed against InvA and YadA. As a molecular marker the PageRuler Prestained Protein Ladder was loaded on the left.

Besides invasin, also the virulence plasmid-encoded YadA protein promotes tight adhesion of *Y. enterocolitica* to host cells [43]. We first investigated expression of the *yadA* gene in response to temperature and found that similar amounts of YadA are produced in all tested *Y. enterocolitica* O:8 and O:3 strains at 37°C (Fig. **5B**, **6B**) whereas no synthesis could be detected at 25°C (data not shown). Furthermore, we analyzed cell adhesion and internalization of *Y. enterocolitica* O:3 strain Y1 grown at 25°C or 37°C in the presence and absence of invasin or YadA (Fig. **7A**), and confirmed production or loss of adhesins in the equivalent *Y. enterocolitica* strains (Fig. **7B**). Deletion of the *invA* gene had no effect on host cell binding, but eliminated the ability of the YeO:3 strains to invade human epithelial cells independently from growth temperature. This phenotype was fully complemented by an *invA* expression plasmid. In contrast, loss of YadA had no effect on host cell invasion and cell adherence at 25°C. However, host cell binding and invasion were significantly reduced when *yadA*-deficient bacteria were grown at 37°C. Overexpression of the *yadA* gene under control of an inducible promoter (P*_BAD_*) complemented this phenotype and increased cell binding and entry levels at 37°C. Even more strikingly, it promoted highly efficient cell adhesion and invasion of bacteria grown at moderate temperature, similar to YeO:8 8081v (Fig. **7A**). Thus, co-expression of both adhesins is required to permit efficient cell binding and internalization of serotype O:3 strains into host cells: YadA is needed to maximize adhesion whereas invasin is necessary to initiate the internalization process.

### Analysis of *invA* expression in *Y. enterocolitica* O:3

**Figure 8:**
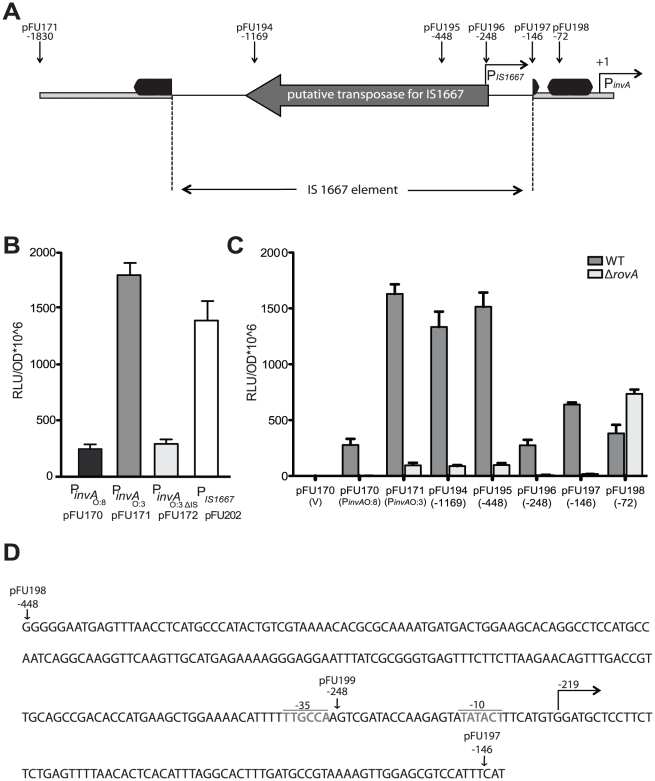
Analysis of *invA* expression in *Y. enterocolitica* O:3. (**A**) An overview of the *invA* promoter region including the IS1667 insertion of *Y. enterocolitica* O:3 strains is shown. The transcriptional start sites of the *invA* gene and from the predicted IS1667-encoded promoter are indicated by broken arrows, the dark boxes indicate the RovA binding sites identified in the homologous *invA* promoter of *Y. pseudotuberculosis*. The thick line represents the *invA* promoter sequence and the thin line illustrates the IS1667 sequence. The arrow indicates the gene encoding the putative transposase of the IS1667 element. Sites used for the upstream deletion constructs are indicated by arrows. The numbers indicate the position of the deletion relative to the transcriptional start site of the *invA* gene. (**B**) Overnight cultures of *Y. enterocolitica* O:3 strain Y1 harbouring the P*_invA_*_O:8_::*luxCDABE* (pFU170), P*_invA_*_O:3_::*luxCDABE* (pFU171), P*_invA_*_O:3ΔIS_::*luxCDABE* (pFU172) and P_IS1667_::*luxCDABE* (pFU202) fusion constructs were diluted (1∶100) and grown in LB at 37°C for four hours and luciferase activity was determined. (**C**) Expression by progressive deletion of the *invA* 5′-regulatory region was analyzed in *Y. enterocolitica* O:3 Y1 and the isogenic *rovA* mutant derivative Y12 harbouring the P*_invAO:3_*::*luxCDABE* fusion. The numbers indicate the 5′ end points of the regulatory region of *invA* from *Y. enterocolitica* O:3 in the fusion constructs relative to the transcriptional start site (+1). The luciferase activity determined from the cultures is given in relative light units (RLU) and represents the mean ± standard deviation of at least three independent experiments. (**D**) Sequence of the 3′-end of the IS1667 inserted into *invA* of *Y. enterocolitica* O:3 at position −143 is shown. The −10 and −35 region of the predicted IS1667-encoded promoter are indicated. Sites used for the upstream deletion constructs are indicated by arrows. The numbers indicate the position of the deletion relative to the transcriptional start site of the *invA* gene.

Our previous experiments clearly demonstrated that the absence of YadA results in low invasiveness of YeO:3 strains at 25°C, despite the presence of invasin. Yet, invasin expression at 37°C is a special feature of serotype O:3 strains, as it is not produced in other previously characterized *Yersinia* strains, e.g. YeO:8 8081v (Fig. **5**) [15] and *Y. pseudotuberculosis* [44]. Therefore, we started to elucidate the molecular mechanisms underlying such differences. First, the *invA* coding and regulatory region of all *Y. enterocolitica* O:3 isolates used in this study were sequenced and an IS1667 element inserted at position −143 of the *invA* promoter was identified (Fig. **8A**). To address whether presence of the IS1667 insertion is restricted to strains of the same geographic region isolated over a relatively short timeframe, we also sequenced the *invA* locus of 22 additional *Y. enterocolitica* O:3 isolates collected from all over the world between 1973 and 2008 (Table **S1**). All tested isolates contained the IS1667 element at the same position within the *invA* regulatory region. To test the influence of the inserted IS element, we compared the activities of the *invA* promoter of YeO:8 8081v (P*_inv_*_O:8_) and YeO:3 Y1 wild-type (P*_inv_*_O:3_) or after deletion of the IS1667 insertion (P*_inv_*_O:3ΔIS_). We found that integration of the mobile element is accompanied with a much stronger expression of the *invA* promoter. As shown in Fig. **8B**, expression of the P*_inv_*_O:3ΔIS_::*luxCDABE* and the P*_inv_*_O:8_::*luxCDABE* fusions were very similar and significantly lower than P*_inv_*_O:3_::*luxCDABE* expression at 37°C. This result is consistent with a western blotting analysis showing that invasin production is considerably higher in the YeO:3 strains than in YeO:8 strain 8081v at 37°C (Fig. **5**). As the *luxCDABE* reporter generates a non-linear and often stronger signal than the relative change in transcription, we also performed a quantitative RT-PCR analysis and observed a 6.5-fold reduction of relative *invA* mRNA levels in the ΔIS1667 mutant YE15 compared to the wild-type strain (Fig. **S3**).

To find out whether higher activation of the *inv*O:3 promoter was due to the insertional inactivation of inhibitory sequences (e.g. H-NS binding sites) or to the presence of specific IS sequences different portions of the *invA* upstream region were deleted and transcription of the P*_inv_*_O:3_::*luxCDABE* fusion in the Y1 wild-type strain was analyzed. High expression of the P*_inv_*_O:3_::*luxCDABE* fusion was obtained with deletion constructs harboring sequences upstream of position −448, whereas P*_inv_*_O:3_ promoter activity was severely reduced with the fusions starting at or downstream from position −248 (Fig. **8C**). This demonstrated that the P*_inv_*_O:3_ activity cannot solely be caused by insertional inactivation of inhibitory sequences, and indicated that an IS-encoded function contributes to P*_inv_*_O:3_ activation. In fact, insertion of the IS1667 sequences from position −448 and −144 upstream of the promoterless *luxCDABE* operon resulted in strong expression of the fusion construct, indicating that an additional promoter (P*_IS1667_*) oriented outward of the IS element drives *invA*_O:3_ expression (Fig. **8B**). In fact, primer extension analyses revealed a strong IS-encoded promoter (P*_IS1667_*) with a −35 region located upstream and the −10 region downstream of position −248. P*_IS1667_* initiated transcription from position −219 with respect to the transcriptional start site of a second promoter (P*_invA_*) located within the *invA* regulatory region (Fig. **8D**, **S4**). P*_invA_* was equal to the *invA* promoter of *Y. enterocolitica* O:8 [15] and exhibited a similar activity when the inserted IS1667 element was deleted (Fig. **8B**).

Since the IS1667 is inserted into the 3′-end of the binding site I of the transcriptional activator protein RovA (Fig. **8A**) [45], [46], we also analyzed whether *invA* expression in *Y. enterocolitica* O:3 strain Y1 is still dependent on RovA. We found that *invA* mRNA levels and the activity of all highly activated P*_inv_*_O:3_::*luxCDABE* fusions starting from position −1830, −1169 and −448 were significantly reduced in the absence of the *rovA* gene, demonstrating that strong enhancement of *invA* expression by the IS-encoded promoter still requires the function of the transcriptional activator protein (Fig. **8C**, **S3**).

**Figure 9:**
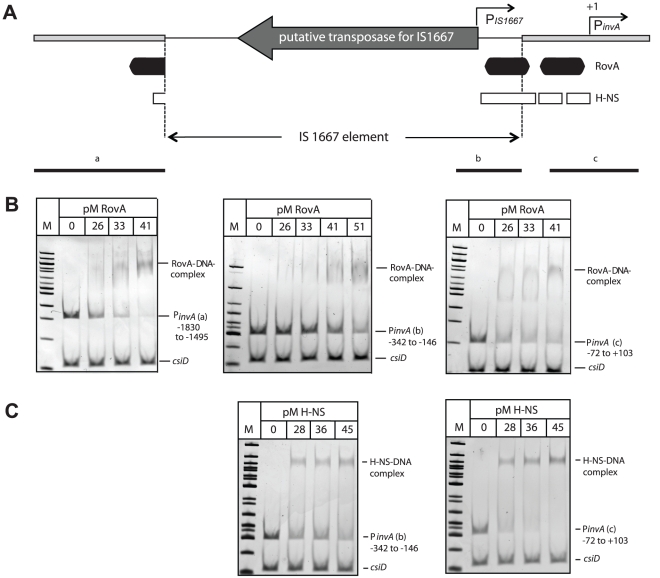
RovA and H-NS binding to the *Y. enterocolitica* O:3 *invA* regulatory region. (**A**) Overview of the *invA* promoter region of *Y. enterocolitica* O:3 strains. The transcriptional start sites of the *invA* promoter and of the predicted IS1667-encoded promoter are indicated by broken arrows. The dark boxes represent the RovA and the white small boxes the H-NS binding sites identified in the homologous *invA* promoter of *Y. pseudotuberculosis*. The thick line represents the *invA* promoter sequence and the thin line illustrates the sequence of the IS1667 element with the putative transposase gene. Fragments used for the band shift experiments are shown as black lines. Competitive gel retardation assays using purified RovA protein (**B**) or purified H-NS (**C**) of *Y. enterocolitica* O:3 strain Y1. DNA fragments comprising different portions of the *invA* regulatory region of Y1 were incubated without or with increasing concentrations of purified RovA or H-NS. The DNA-protein complexes were separated on a 4% polyacrylamide gene, a molecular weight standard 100 bp ladder was loaded on the left. The higher molecular weight protein-DNA complexes are marked by an arrow and the positions of the non-shifted and control fragments are indicated.

Previous footprint analysis revealed that RovA interacts with two distinct binding sites of the *Y. pseudotuberculosis invA* promoter [46], and sequence homology as well as RovA band shift analysis indicated that similar binding sites are also recognized by RovA in the *invA* regulatory region of YeO:8 8081v [45]. RovA-binding site I was partially destroyed by the insertion of the IS1667 element in the *invA*_O:3_ promoter (Fig. **9A**). However, band shift analysis with purified recombinant RovA and different DNA fragments of the *invA* promoter region demonstrated that RovA still interacts specifically with RovA sequences upstream of the IS1667 element containing major parts of binding site I (Fig. **9B**). Interestingly, RovA was also found to specifically interact with fragments harboring the 3′-end (−342 to −146) of the integrated mobile element, although a slightly higher concentration was required for RovA-DNA complex formation. This demonstrated that this portion of the IS1667 element includes sequences, which are also preferentially recognized by RovA.

It is very likely that RovA is needed to alleviate H-NS-mediated repression at sites located downstream of the IS1667 insertion (Fig. **9A**) to permit maximal transcription of the *invA* promoter. To test this hypothesis, we also studied the interaction of H-NS with different fragments of the *inv*O:3 promoter region. As shown in Fig. **9C**, H-NS was able to preferentially interact with a fragment harboring the 3′-portion of the IS1667 element (−342 to −146), but the affinity was slightly lower compared to H-NS binding to the *invA* promoter fragment (−72 to +103). This strongly suggests that RovA is still required to eliminate H-NS mediated repression to allow optimal expression of invasin by the P*_IS1667_* and the P*_invO:3_* promoter.

### Enhanced RovA production in *Y. enterocolitica* O:3 strains

Requirement of RovA for *invA* transcription in *Y. enterocolitica* O:3 at 37°C was unexpected as it has been shown that *rovA* expression in *Y. enterocolitica* O:8 and *Y. pseudotuberculosis* strains is strongly thermoregulated and only expressed at moderate temperatures [44], [47]. The RovA protein was found to act as a thermosensor which undergoes a conformational change upon a temperature shift from 25°C to 37°C. This thermo-induced conformational change reduces the DNA binding activity of the regulatory protein and renders it more susceptible to proteolysis by the Lon protease [18]. As a result, RovA activation of *invA* expression is abolished at 37°C. Although RovA was shown to activate *invA* expression in *Y. enterocolitica* O:3 (Fig. **8C**), invasin expression does not appear to be strongly temperature-regulated compared to other *Yersinia* strains (Fig. **5**, **6B** [15], [44]).

**Figure 10:**
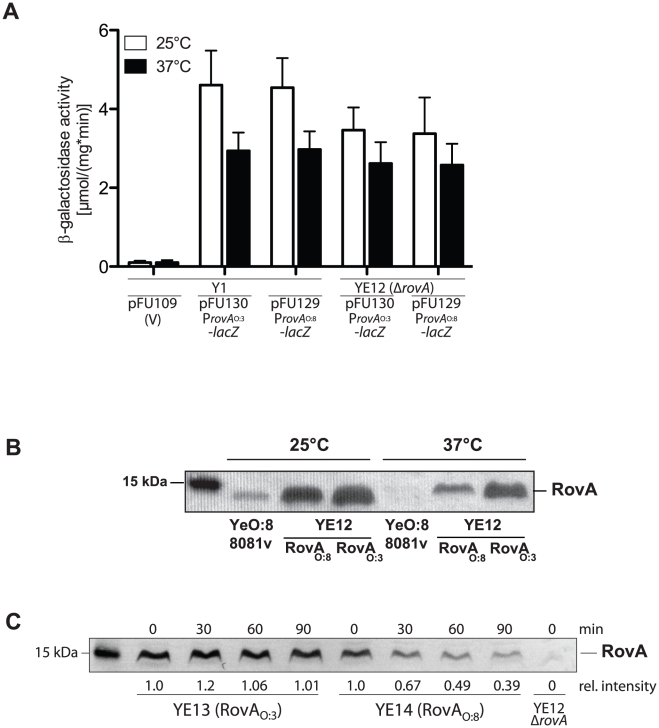
Analysis of RovA production and stability in *Y. enterocolitica* O:3. (**A**) *Y. enterocolitica* strains Y1 and the isogenic *rovA* mutant of Y1 (YE12) harboring plasmids encoding the promoterless *lacZ* gene or the P*_rovA_*_O:8_-*lacZ* or P*_rovA_*_O:3_-*lacZ* fusions were grown at 25°C and 37°C overnight. The beta-galactosidase activity determined from the cultures is given in µmol min^−1^ mg^−1^ and represents the mean ± standard deviation of at least three independent experiments. (**B**) A *Y. enterocolitica* O:3 Δ*rovA* mutant strain (YE12) harboring the *rovA* encoding plasmids pFU119 (*rovA*_O:3_) or pFU138 (*rovA*_O:8_) and YeO:8 strain 8081v were grown overnight at 25°C and 37°C. Whole cell extracts were prepared from the cultures, separated on SDS-polyacrylamide gels and analyzed by western blotting using polyclonal antibodies directed against RovA. As a molecular marker the PageRuler Prestained Protein Ladder was loaded on the left. (**C**) Isogenic *Y. enterocolitica* strains YE13 and YE14 expressing the RovA wildtype protein or the RovA_S98P_ derivative were grown to exponential phase (OD_600_ = 0.6–0.7) at 37°C before gentamicin (50 µg ml^−1^) and tetracycline (50 µg ml^−1^) were added. The cultures were incubated at 37°C for additional 90 min. Aliquots of the cultures were removed at the indicated times thereafter, whole cell extracts for identical numbers of bacteria were prepared and intracellular RovA was visualized by western blotting.

To better understand the different control mechanisms, *rovA* expression in the different *Y. enterocolitica* isolates was analyzed. We found that all YeO:3 isolates produced very high levels of RovA at 25°C and 37°C; whereas no RovA was detected at 37°C in other *Yersinia* strains, e.g. YeO:8 strain 8081v and *Y. pseudotuberculosis* (Fig. **5**, data not shown [44], [47]). Expression analysis of the P*_rovA_*_O:3_::*lacZ* and P*_rovA_*_O:8_::*lacZ* fusions revealed that both *rovA* promoters are not auto-activated and are either not or only very weakly dependent on the temperature (Fig. **10A**, **S6**). Next, we addressed whether thermo-sensing and proteolysis varies between the RovA_O:8_ and RovA_O:3_ proteins. We introduced low-copy plasmids carrying the *rovA*O:3 gene of YeO:3 Y1 or the *rovA*O:8 gene of YeO:8 8081v into a *Y. enterocolitica* O:3 *rovA* mutant strain (YE12) and compared RovA levels after growth at 25°C and 37°C (Fig. **10B**). Almost identical levels of the RovA proteins were detected at 25°C. However, significantly lower amounts of the RovA_O:8_ protein were visible at 37°C, while RovA_O:3_ concentrations remained almost the same (Fig. **10B**). This strongly suggested that post-transcriptional mechanisms controlling RovA levels must be different in YeO:3 strains. To test this hypothesis, we sequenced the *rovA* locus of all 49 available *Y. enterocolitica* O:3 strains (Table **S1**). We found that the *rovA* genes of 45 strains, including all isolates tested in this study vary from *rovA* of other *Y. enterocolitica* serotypes by a single point mutation in codon 98, resulting in a P98S change in the amino acid sequence of the translated regulatory protein. To find out whether this substitution affects function of RovA as a thermosensor, we overexpressed and purified RovA of YeO:8 8081v and YeO:3 Y1 and compared their DNA-binding capacity at 25°C and 37°C (Fig. **S5**). However, interaction of both RovA variants with DNA fragments of the *invA* regulatory region was still temperature-dependent. Significantly more of both RovA proteins was required at 37°C for RovA-DNA complex formation, indicating that the thermosensing function is not severely affected by the P98S exchange. Next, we addressed thermo-dependent susceptibility of the RovA variants to degradation by the Lon protease. To this aim, we reintegrated a copy of the *rovA*_O:3_ or *rovA*_O:8_ gene into the genome of a *rovA* deficient YeO:3 strain (YE12) and performed stability assays. Identical amounts of RovA_O:3_ were still visible 90 min after protein biosynthesis was stopped (Fig. **10C**). In contrast, the RovA_O:8_ protein was rapidly degraded at 37°C, and significantly lower amounts of the regulatory protein were detectable 90 min after cessation of protein synthesis.

### Influence of higher RovA and invasin levels in YeO:3 on invasion and virulence

**Figure 11:**
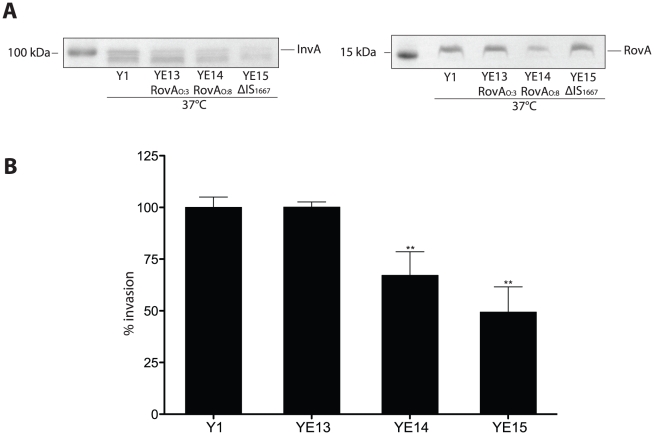
Influence of enhanced invasin and RovA levels on *Y. enterocolitica* O:3 host cell invasion. (**A**) Whole cell extracts were prepared from the cultures, separated on SDS-polyacrylamide gels and analyzed by western blotting using polyclonal antibodies directed against InvA and RovA. As a molecular marker the PageRuler Prestained Protein Ladder was loaded on the left. (**B**) YeO:3 strains Y1 (wt), YE13 (*rovA*O:3_S98_), YE14 (*rovA*O:8_P98_) and YE15 (*rovA*O:3_ΔIS1667_) were grown at 37°C. Approximately 10^6^ bacteria were centrifugated onto 10^4^ HEp-2 cells. Total numbers of intracellular bacteria were determined and are expressed relative to the invasion rate of YeO:3 strain Y1 defined as 100%. Each value represents the mean of at least three different assays done in triplicate. Data were analyzed by the students t test, **, significantly different from Y1 or YE13 with P<0.001.

To test the effect of the IS1667 insertion in the *invA* promoter and the more stable RovA_O:3(S98)_ variant on host cell invasion, we compared the amount of produced invasin and RovA in YeO:3 strains YE13 (*rovA*_O:3_), YE14 (*rovA*_O:8_) and YE15 (P*_inv_*_O:3ΔIS_) (Fig. **11A**) and investigated the efficiency of these bacteria to enter HEp-2 cells (Fig. **11B**). High levels of invasin were detectable in YE13, whereas the instable RovA variant and deletion of the IS1667 element produced lower amounts of invasin, leading to a significant reduction of invasiveness into human epithelial cells.

**Figure 12:**
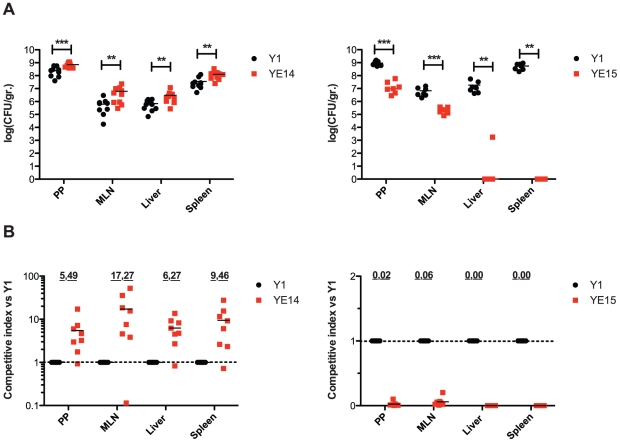
Influence of enhanced invasin and RovA levels on *Y. enterocolitica* O:3 virulence. (**A**) BALB/c mice were co-infected via the orogastric route with 5×10^8^ bacteria in an inoculum comprised of an equal mixture of YeO:3 strains Y1 (wt, *rovA*_O:3_) and YE14 (*rovA*_O:8_), or Y1 (wt, *rovA*_O:3_) and YE15 (*rovA*O:3_P*inv*ΔIS_). Three days post infection, the mice were sacrificed and the numbers of surviving bacteria in the liver, spleen, mesenterial lymph nodes (MLN), and Peyer's patches (PP) were determined as described in *Material and Methods*. Data are presented as a scatter plot of numbers of cfu per gram of organ as determined by counts of viable bacteria on plates. Each spot represents the cfu count, in the indicated tissue samples from one mouse. The levels of statistical significance for differences between test groups were determined by the Mann-Whitney-test. Stars indicate results that differed significantly from those of Y1 with ** (P<0.01), and *** (P<0.001). (**B**) Data are graphed as competitive index values for the tissue samples from one mouse. The bars represent the means of the competitive index values. A competitive index score of 1 denotes no difference in the virulence compared to Y1. Underlined scores denote where statistically significant differences were observed. The two strains Y1 and Y15 used for competition assays were differentially marked with antibiotics resistances on plasmids.

It was previously shown that invasin and RovA are important to invade the intestinal epithelium by *Y. enterocolitica* O:8 early after infection. The *rovA*-deficient mutants were found to be attenuated in the ability to reach and/or replicate in the deeper tissues and organs and induce a milder inflammation of the Peyer's patches [48], whereas the LD_50_ values of the wild-type and the *invA* mutant were essentially identical but the colonization of the host tissues was delayed [4]. In order to determine whether higher invasin and RovA levels in *Y. enterocolitica* O:3 also affect pathogenesis, we tested the virulence of wild-type and mutant strains in the murine infection model. First, single strain infections were performed and bacterial colonization of Peyer's patches (PPs), mesenteric lymphnodes (MLNs), liver and spleen was assessed. Since only minor differences could be highlighted (data not shown), we performed co-infection experiments to determine whether presence of the wild-type affects the ability of the mutants to colonize tissues in a single host. This minimizes inherent inter-animal biological variations and can expose even subtle differences of the biological fitness and virulence, e.g. in the kinetics of infection. BALB/c mice were orally infected with 5×10^8^ bacteria in an inoculum comprised of an equal mixture of (i) the parental Kan^S^ wild-type strain Y1 (*rovA*_O:3(S98)_) and the Kan^R^ mutant strain YE14 (*rovA*_O:8(P98)_) or (ii) Y1 and YE15 (P*_inv_*_O:3ΔIS_) harboring a stable vector which only differs in its antibiotic resistance cassette to establish the ability to discriminate strains. Three days after infection, mice were dissected and the numbers of bacteria present in the PPs, MLNs, liver or spleen were determined (Fig. **12**). The results of the infection showed that both, the parental (YE13 *rovA*_O:3(S98)_) and the *rovA*_O:8(P98)_ mutant strain (YE14) are capable of establishing an infection, but considerably higher numbers of bacteria encoding the less stable RovA_O:3(P98)_ variant from YeO:8 (YE14) were recovered from all dissected tissues. About 2- to 10-fold more bacteria of this strain were isolated from the lymphatic tissues or the organs (Fig. **12A**) compared to the parental strain YE13 (*rovA*_O:3(S98)_). Also comparison of the relative virulence ratio (Fig. **12B**) and calculation of the competitive index of the mutant relative to the wild-type strain (Fig. **12C**) indicated that higher concentrations of RovA during mouse infections at 37°C are disadvantageous for the colonization and multiplication of YeO:3 in the organs. In contrast, significantly lower numbers of strain YE15 lacking the IS1667 element in the *invA* promoter region were isolated. About 10–20 times less bacteria were recovered from the PP and MLNs (**Fig. 12A**). The difference in the dissemination of the bacteria was even more striking. The IS1667 deletion strain YE15 was strongly attenuated in its ability to reach deeper tissues. Only in some occasions it reached the liver and spleen, but the bacterial load of the mutant in the liver and spleen was always significantly lower compared to wild-type (Fig. **12**). In summary, these data strongly indicates that high invasin expression levels during the course of an infection combined with a fine-tuned control of the virulence regulator RovA are advantageous for YeO:3 virulence in mice.

## Discussion

**Figure 13:**
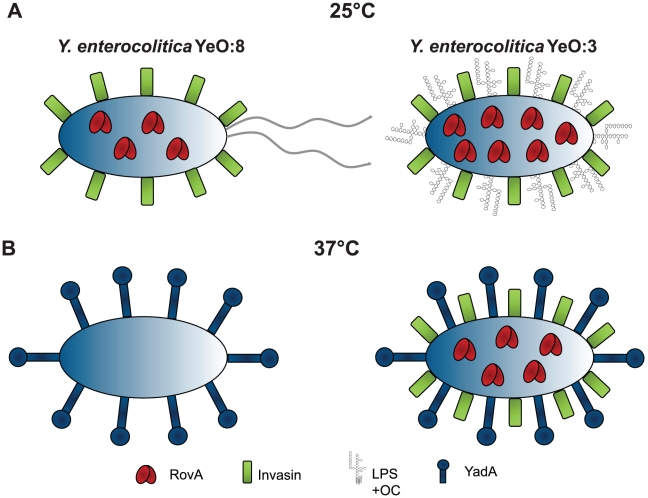
Comparison of *Y. enterocolitica* O:3 and O:8 mediated temperature regulated control of host cell invasion. Model of virulence factor expression of *Y. enterocolitica* O:3 and O:8 in response to temperature. (**A**) At moderate temperature, *rovA* expression is induced in *Y. enterocolitica* O:8 which leads to activation of invasin expression. Furthermore, flagella production is activated and enhances host cell contact, and LPS molecules are synthesized which do not interfere with invasin function. This leads to an efficient internalization of the serotype O:8 strains after growth at environmental temperatures. At 37°C, RovA is rapidly degraded resulting in downregulation of invasin. In addition, flagella and O-antigen production is repressed, whereas synthesis of the adhesin YadA is induced which allows efficient adhesion, but no internalization into epithelial cells. (**B**) *Y. enterocolitica* O:3 produce similar and significantly higher amounts of invasin at environmental and body temperature due to an IS insertion into the *invA* upstream region and a stable RovA activator protein both abolishing H-NS mediated repression. However, internalization into host cells is strongly reduced at 25°C due to steric hindrance by the unique O-antigen and repression of YadA which strongly enhances and stabilizes host cell interactions at 37°C. LPS+OC: lipopolysaccharides with O-antigen and outer core.

The ability of *Y. enterocolitica* to bind and invade into host cells is essential for pathogenesis and persistance in its human host. Results of the present investigation highlight important differences in the adhesion properties between serotype O:3 strains (responsible for more than 70% of human yersiniosis cases) and other *Y. enterocolitica* serotypes, e.g. serotype O:8, whose pathogenicity has been extensively investigated. Comparative analysis of cell binding properties demonstrated that the same repertoire of virulence factors is implicated in host cell binding in the serotype O:3 isolates, but their interplay and expression profile in response to environmental signals is significantly different from O:8 strains (Fig. **13**).

We show that synthesis of the primary internalization factor invasin is highly activated and nearly constitutive in all tested *Y. enterocolitica* O:3 strains. This is in contrast to O:8 serotypes in which invasin synthesis is repressed at 37°C due to H-NS mediated silencing and rapid degradation of the *invA* activator protein RovA. Interestingly, a previous study also reported that *invA* expression of a serotype O:9 strain was higher than in serotype O:8, but it was still significantly reduced at 37°C [49]. Constitutive expression of the *invA* gene in the O:3 strains was acquired by an IS1667 insertion into the *invA* regulatory region harbouring RovA and H-NS binding sites. Gene activation by transposons has been described for other genetic systems but the induction mechanism of the *Y. enterocolitica* O:3 *invA* gene seems distinct from previously reported systems. Transposable elements usually activate gene expression by replacing a negative regulatory element or through introduction of promoter elements [50], [51]. One of the best-characterized examples of transposon-mediated gene activation is the beta-glucoside (*bgl*) operon of *E. coli*. This system is usually repressed but can be activated by IS insertions up- or downstream of the promoter in either orientation relieving H-NS repression [52], [53]. However, deletion analysis revealed that transposon-mediated *invA* activation in *Y. enterocolitica* O:3 is not solely due to disruption of the inhibitory H-NS binding sites, but also requires an IS-specific activating element. One recent study revealed a novel transposon-mediated gene activation mechanism. An IS5 insertion at a single site and in only one orientation was found to activate expression of the *glpFK* operon in a *crp* background [54]. A short sequence at the 3′ end of the IS5 transposon, including a permanently bent polyA-tract and an IHF binding site, was shown to be required for *glpFK* induction. This shows that unique sequences within a mobile element can act as an enhancer or gain an activator binding function sufficient to activate close promoters. In this study we found that IS1667-promoted activation of *invA* expression in *Y. enterocolitca* O:3 at 25°C and 37°C is largely dependent on the presence of an IS1667-generated promoter and alternative RovA (activator) and H-NS (silencer) binding sites. RovA of YeO:8 was previously shown to activate *invA* expression only at moderate temperatures through antirepression of H-NS-mediated silencing [45]. A temperature upshift to 37°C, however, results in a conformational change within RovA that strongly reduces the DNA-binding capacity of the regulator. It has been previously shown that the apparent dissociation constant (K_d_) of the thermoregulated RovA protein of *Y. pseudotuberculosis* is about four-fold increased upon a temperature shift from 25°C to 37°C [18]. Furthermore, it was found that the temperature upshift renders the RovA protein more susceptible to degradation by the Lon and ClpP proteases [18]. Comparable studies with the RovA protein of YeO:8 8081v demonstrated similar properties and identical function as an intrinsic thermosensor (F. Uliczka, unpublished data). Here, we found that a single proline to serine exchange at position 98 (P98S) increases the stability of YeO:3 RovA without affecting the thermosensing ability of the protein. As a consequence, significantly higher RovA concentrations are present within the bacteria and this is sufficient to compensate for the thermo-induced reduction of RovA DNA binding. As YeO:3 strains originate mainly from boars and pigs with a higher body temperature of about 39°–40°C, a more temperature-stable RovA variant might be advantageous for persistence in these animals. According to our proposed structure model of RovA [55] the amino acid P98 is located in a surface exposed loop structure and is as such easily accessible for the proteases. How the P98S mutation affects proteolytic degradation is not yet clear. However, comparative CD spectroscopy of purified RovA_O:8(P98)_ and a RovA_O:3(S98)_ variant of *Y. pseudotuberculosis* indicated that no major structural changes are induced by this amino acid substitution (N. Quade, unpublished results). Furthermore, proteolysis is drastically reduced but not completely blocked by the P98S mutation as a slightly higher concentration of the regulatory protein was detected in a *Yersinia lon* mutant strain.

In summary, a more stable RovA variant (RovA_O:3(S98)_) and an IS1667 insertion in the *invA* promoter region, providing an additional promoter followed by slightly weaker RovA and H-NS binding sites, allow high expression levels of invasin in YeO:3 strains at 37°C. How these different properties influence pathogenesis is not fully clear, but first experiments addressing host cell invasion and colonization of YeO:3 in the mouse model revealed that loss of the IS1667 element reduced host cell entry and had a severe effect on the infection process in mice. Colonization of the PPs and the MLNs by YeO:3 strain YE15 (P*_inv_*_O:3ΔIS_) was significantly reduced and only occasionally these bacteria were able to reach deeper organs in co-infection experiments. This indicates that high levels of invasin are more advantageous and/or important for YeO:3 to initiate a successful infection than for YeO:8 in mice. In fact, a YeO:8 8081v *invA* mutant strain shows a delayed but still efficient colonization of deeper tissues [4], [56].

In contrast to invasin, loss of the RovA regulator in YeO:8 8081v leads to a 70-fold increase of the LD_50_ and causes a much more severe alteration of the infection kinetics, e.g. penetration of the Peyer's patches and mesenterial lymph nodes was much more reduced, and dissemination into liver and spleen was abolished [56]. Interestingly, significantly higher numbers of bacteria could be detected in lymphatic tissues and organs of mice when the unstable variant RovA_O:3(S98)_ was expressed by YeO:3. This strongly suggests that elevated RovA levels, although they lead to higher amounts of invasin are disadvantageous for the colonization of the organs in mice. Microarray analysis to define the RovA regulon of *Y. enterocolitica* in YeO:8 revealed 40 genes to be activated and 23 repressed by RovA [57]. Among the RovA-repressed loci are several metabolic genes, e.g. permeases for glutamine, glutamate and aspartate) and their upregulation due to reduced RovA levels at 37°C might be important for the biological fitness and survival in host tissues during infection in mice. A more stable but still thermo-sensitive RovA variant, as found in YeO:3 strains (RovA_O:3(P98)_), would allow similar regulatory control over virulence and metabolic genes in pigs and boars with a higher body temperature (39°C–40°C). In order to test whether the IS1667 insertion in the *invA* promoter region and the RovA_O:3(S98)_ variant reflects an optimal adaptation to these host organisms we are currently establishing a pig infection model.

Although high levels of invasin are produced by YeO:3 strains at moderate growth temperatures, cell invasion was either not initiated or very inefficient when the bacteria were pregrown at 25°C. This is in strong contrast to other *Y. enterocolitica* serotypes or *Y. pseudotuberculosis* isolates which enter host cells with their highest efficiency when cultured at moderate temperatures. Previous analyses showed that induced flagellar-dependent motility is required for efficient invasion of YeO:8, but flagella production of this pathogen is repressed at 37°C [34]. Flagella are needed to ensure migration of the bacteria to host cells, but are not essential for the invasion process once the bacteria contact the mammalian cells. Motility assays and electron microscopy revealed that flagellated and motile strains of *Y. enterocolitica* O:3 strains can be isolated from the intestinal tract of a mouse, but they rapidly loose their motility and become aflagellated during growth under standard laboratory conditions. As a result, YeO:3 strains are less invasive than other motile serotypes *in vitro*, but cell entry could be improved upon artificial host cell contact by centrifugation.

However, when the bacteria were pregrown at 25°C, YeO:3 uptake after host cell contact is still less efficient compared to YeO:8 or other serotypes, indicating that other factors repress invasin-mediated internalization at moderate temperatures or enhance cell entry at 37°C. *Y. enterocolitica* isolates grown at room temperature generally express LPS with O-ag, whereas only very small amounts of O-ag are present in bacteria grown at 37°C [33], [58]. The O-ag of YeO:8 is required for full virulence and plays a major role in pathogen-host interplay by affecting the expression and function of other *Yersinia* virulence factors, e.g. absence of the O-ag reduced *invA* expression and internalization into HeLa cells [31]. In contrast, O-ag deficient YeO:3 rough mutants are more efficiently internalized by human epithelial cells. Furthermore, no reduction of *invA* expression was observed in the rough mutants at 37°C when O-ag expression is fully repressed. Unlike other *Yersinia* serotypes and other Gram-negative bacteria, the YeO3 O-ag forms a long homopolymer that is linked together with the OC hexasaccharide to the inner core forming a unique branched LPS structure. Its formation was previously shown to prevent proper function of some small size outer membrane proteins. For instance, O-ag was shown to inhibit serum resistance indirectly by masking the adhesin Ail from complement regulator C4bp binding [59]. Therefore, reduced O-ag density in YeO:3 at 37°C is very likely diminishing sterical hindrance thus allowing better access and host cell receptor binding by surface adhesins such as invasin and YadA (Fig. **13**).

In fact, besides invasin, also production of the adhesin YadA is required to promote efficient uptake of YeO:3. The virulence plasmid encoded trimeric YadA protein is highly and exclusively expressed at 37°C, and forms a capsule-like, fibrillar matrix covering the bacterial surface [60]. YadA of *Y. enterocolitica* O:8 strains has been shown to promote tight binding to extracellular matrix proteins such as collagen and laminin, but it does not contribute to epithelial cell entry compared to invasin [19], [61]. In fact, at 37°C when YadA is highly expressed but no or only very low levels of invasin are produced by YeO:8, no internalization of the bacteria is initiated (Fig. **3B**, **6A**). Internalization of YeO:3 at 37°C also seems to be exclusively mediated by invasin as an *invA* mutant is unable to enter host cells. Yet, YadA synthesis is not dispensable, as its absence in a *yadA* mutant or during growth at 25°C results in a significantly lower cell adhesion and uptake rate even in the presence of high amounts of invasin, whereas *yadA* expression by an inducible promoter at 25°C leads to strong adhesion and efficient invasion of YeO:3 similar to YeO:8. YadA seems to be required to guarantee tight and efficient host cell binding which then in turn leads to a more efficient invasin-mediated uptake. Both invasin and YadA promote direct or indirect binding to beta 1 integrins [13], [62]. High affinity binding and ligand-induced beta 1-integrin-clustering by invasin are required for efficient uptake by this host cell receptor family [63], [64]. However, invasin of *Y. enterocolitica* does not contain a self-association domain mediating receptor-clustering and uptake in contrast to invasin of *Y. pseudotuberculosis* [65]. It is therefore tempting to speculate that co-expression of the somewhat longer cell surface adhesin YadA which promotes binding to ECM molecules bound to beta 1 chain integrins promotes or enhances intimate direct interaction of invasin and subsequent internalization (Fig. **13**).

In summary, results in the present investigation provide evidence that even small variations between virulence factors and regulators are responsible for the substantial difference in host cell interactions of *Y. enterocolitica* serotype O:3 in comparison to other *Y. enterocolitica* serotypes. Serotype O:3 specific variations in the surface molecule expression pattern imply that this *Y. enterocolitica* subspecies varies in its dynamic capacity to adapt to changing environments and individual niches within the host. A particular repertoire of host interaction genes may confer a survival advantage or pathogenic potential in a specific microenvironment. Thus, an individual subspecies may be better adapted for survival in a particular host or host site, e.g. human gastrointestinal tract or oral cavities of swine (e.g. tongue and tonsils).

## Materials and Methods

### Ethics statement

All animal work was performed in strict accordance with the German regulations of the Society for Laboratory Animal Science (GV-SOLAS) and the European Health Law of the Federation of Laboratory Animal Science Associations (FELASA). The protocol was approved by the Niedersächsisches Landesamt für Verbraucherschutz und Lebensmittelsicherheit: animal licensing committee permission no. 33.9.42502-04-055/09. All efforts were made to minimize suffering.

### Bacterial strains, cell culture, media and growth conditions

The strains used in this study are listed in **Table 1**. Overnight cultures of *E. coli* were routinely grown at 37°C, *Yersinia* strains were grown at 25°C or 37°C in LB (Luria-Bertani) broth. The antibiotics used for bacterial selection were as follows: ampicillin 100 µg/ml, chloramphenicol 30 µg/ml, kanamycin 50 µg/ml, gentamicin 50 µg/ml and tetracycline 10 µg/ml. For infection experiments, bacteria were grown at 25°C or 37°C, washed and diluted in PBS prior to infection.

**Table 1:** Bacterial strains and plasmids.

Strains, Plasmids	Description	Source and reference
**Bacterial strains**		
*E. coli* K-12		
DH101beta	F^−^ *endA1 recA1 galE15 galK16 nupG rpsL ΔlacX74*	Invitrogen
	φ80*lac*ZΔM15 *araD139* Δ(*ara,leu*)7697 *mcrA*	
	Δ(*mrr-hsdRMS-mcrBC*) λ^−^	
S17-1 λpir	Tp^r^ Sm^r^ *recA*, *thi*, *pro*, *hsdR* ^−^ *M* ^+^ RP4:2-Tc:Mu:Km Tn7	[73]
	λpir	
BL21 CodonPlus	*F^−^ ompT hsdS*(r_B_^−^m_B_^−^) *dcm^+^* Tet^r^ *gal*λ (*DE3*) *endA*	Stratagene
(DE3)-RIL	Hte [*argU ileY leuW* Cam^r^] (DE3)- RIL	
BL21 λDE3	F^−^ *ompT gal dcm lon hsdSB*(r_B_^−^ m_B_^−^) *gal* λDE3	[74]
KB4	BL21 λDE3 *stpA hns hha*	Katja Böhme
*Y. enterocolitica*		
YeO3	6471/76 serotype O:3, patient isolate, wild-type	[75]
YeO3-OC	6471/76, Δ(*wzx-wbcQ*), outer core negative	[42]
	derivative of 6471/76	
YeO3-OCR	spontaneous rough derivative of YeO3-OC	[42]
YeO3-R2	spontaneous rough derivative of YeO3	[30]
Ye 8081v	bioserotype 1A/O:8, patient isolate, wild-type	[15]
4620	bioserotype 3/O:9, patient isolate, wild-type	A. Fruth
3056	bioserotype 3/O:5,27, patient isolate, wild-type	A. Fruth
Y11	bioserotype 4/O:3, patient isolate, wild-type	A. Rakin
Y1	bioserotype 4/O:3, patient isolate, wild-type	E. Strauch
Y2	bioserotype 4/O:3, patient isolate, wild-type	E. Strauch
Y3	bioserotype 4/O:3, patient isolate, wild-type	E. Strauch
Y4	bioserotype 4/O:3, patient isolate, wild-type	E. Strauch
Y5	bioserotype 4/O:3, patient isolate, wild-type	E. Strauch
Y8	bioserotype 4/O:3, patient isolate, wild-type	E. Strauch
Y32	bioserotype 4/O:3, patient isolate, wild-type	E. Strauch
Y33	bioserotype 4/O:3, patient isolate, wild-type	E. Strauch
Y34	bioserotype 4/O:3, patient isolate, wild-type	E. Strauch
YE01	Y11, Δ*rovA*, Cm^R^	This study
YE12	Y1, Δ*rovA*, Cm^R^	This study
YE13	YE12, P*_rovAO:3_*::*rovA*_S98_, Cm^R^ Kn^R^	This study
YE14	YE12, P*_rovAO:3_*::*rovA*_P98_, Cm^R^ Kn^R^	This study
YE15	Y1, P*_invA_*ΔIS1667	This study
YE18	Y1, Δ*yadA*, Tet^R^	This study
YE21	Y1, Δ*invA*, Kn^R^	This study
**Plasmids**		
pASKIBA43+	overexpression vector, Ap^R^	IBA
pBAD33	overexpression vector, Cm^R^	[76]
pBADmycA-HisA	overexpression vector, Ap^R^	Invitrogen
pET28a	overexpression vector, Kn^R^	Novagen
pFU32	ColE1, promoterless *luc*, Tet^R^	lab collection
pFU49	pBAD33, *invA* ^+^_O:3_, Cm^R^	This study
pFU99	pSC101*, promoterless *lacZ*, Cm^R^	lab collection
pFU100	R6K, mobRP4, promoterless *luxCDABE*, Cm^R^	lab collection
pFU102	pFU100, *rovA*::Cm^R^	This study
pFU109	pSC101*, promoterless *lacZ*, Kn^R^	lab collection
pFU114	pFU100, ‘*invA*’ (nt 40–339), Cm^R^	This study
pFU119	pFU109, P*_rovAO:3_*::*rovA*_O:3_, Kn^R^	This study
pFU129	pFU109, P*_rovAO:8_*::*lacZ*, Kn^R^	This study
pFU130	pFU109, P*_rovAO:3_*::*lacZ*, Kn^R^	This study
pFU138	pFU119, P*_rovAO:3_*::*rovA*_O:8_, Kn^R^	This study
pFU156	pET28a, *rovA^+^*_O:8_, Kn^R^	This study
pFU157	pET28a, *rovA^+^*_O:3_, Kn^R^	This study
pFU167	pFU102, *rovA*::Cm^R^, *sacB*	This study
pFU170	pSC101*, P*_inv_*_O:8_::*luxCDABE*, Kn^R^	This study
pFU171	pSC101*, P*_inv_*_O:3_::*luxCDABE*, Kn^R^	This study
pFU172	pSC101*, P*_inv_*_O:3ΔIS_::*luxCDABE*, Kn^R^	This study
pFU175	pSC101*, promoterless *luxCDABE*, Kn^R^	lab collection
pFU182	pBAD33, *invA* ^+^_O:8_, Cm^R^	This study
pFU184	pFU119, P*_rovAO:3_*::*rovA*_O:3_, Kn^R^	This study
pFU185	pFU138, P*_rovAO:3_*::*rovA*_S98P_, Kn^R^	This study
pFU187	pFU167, *yadA*::Tet^R^	This study
pFU188	pBADmycA-HisA, *yadA* ^+^_O:3_, Ap^R^	This study
pFU190	pFU167 P*_invAO:3_* (−1887 to −1627/−249 to −1), Tet^R^	This study
pFU194	pFU175, −1169 bp *invA* upstream region, Kn^R^	This study
pFU195	pFU175, −448 bp *invA* upstream region, Kn^R^	This study
pFU196	pFU175, −248 bp *invA* upstream region, Kn^R^	This study
pFU197	pFU175, −146 bp *invA* upstream region, Kn^R^	This study
pFU198	pFU175, −72 bp *invA* upstream region, Kn^R^	This study
pFU199	pASKIBA43+, *hns^+^*_O:3_, Ap^R^	This study
pFU201	pSC101*, P*_inv_*_O:3_(−248 to −146)-*luxCDABE*, Kn^R^	This study
pFU202	pSC101*, P*_inv_*_O:3_(−448 to −146)-*luxCDABE*, Kn^R^	This study
pFU213	pFU114, ‘*invA*’ (nt 40–339), Kn^R^	This study
pFU220	pBAD33, *yadA* ^+^_O:3_, Cm^R^	This study
pJE4	R6K, *sacB^+^*, Kn^R^	J. Eitel
pZA31luc	expression vector, p15A, P*_LtetO-1_*, Cm^R^	[77]
pZS*24MCS	expression vector, pSC101*, P*_lac/ara-1_*, Kn^R^	[77]

Human HEp-2 cells were cultured in RPMI 1640 media with GlutaMAX (Invitrogen) supplemented with 7.5% newborn calf serum (Sigma Aldrich) at 37°C in the presence of 5% CO_2_. Human Caco-2 cells were grown in DMEM/HAM's F-12 (Biochrom) supplemented with 10% FBS Superior (Biochrom).

### DNA manipulations and construction of plasmids

All DNA manipulations, PCR, restriction digestions, ligations and transformations were performed using standard techniques as described previously [66], [67]. Plasmids used in this study are listed in **Table 1**, and primers are given in **Table S2**.

Plasmids pFU49 (*invA*_O:3_) and pFU182 (*invA*_O:8_) were constructed by amplification of the *invA* gene from genomic DNA of YeO:3 Y11 and YeO:8 8081v with primers II40/II42 and the PCR-derived fragments were subsequently integrated into the *Sac*I/*Sal*I sites of pBAD33. For the overexpession of RovA_O:8_ the *rovA* gene was amplified from genomic DNA of YeO:8 8081v with primers II417/II418 and the generated fragment was inserted into the *Nco*I/*Xho*I sites of pET28a, generating pFU156. Plasmid pFU157 was obtained by QuikChange mutagenesis of pFU156 using primer II375/II376. Plasmid pFU199 was constructed by inserting a *hns* ^+^_O:3_ fragment amplified with primers II726/II727 into the *EcoR*I/*Sal*I sites of pASKIBA43+. For the construction of pFU220 a *Bam*HI/*Sal*I fragment of pFU188 containing the *yadA* gene was integrated into pBAD33. pFU188 was obtained by insertion of a PCR fragment amplified with primers II517/II518 from genomic DNA of YeO:3 Y11 into the *Nco*I/*Sal*I sites of pBAD/Myc-HisA.

Plasmids pFU170 and pFU171 encoding the P*_invAO:8_*::*luxCDABE* and P*_invAO:3_*::*luxCDABE* reporter fusions were generated by insertion of a PCR fragment amplified with primers II177/II178 from genomic DNA of YeO:8 8081v or YeO3 Y11 in the *Bam*HI/*Sal*I sites of pFU175. To construct plasmid pFU172, carrying the P*_invAO:3__ΔIS_*::*luxCDABE* fusion, two PCR fragments amplified with primer pairs II177/II179 and II180/II178 were first ligated with their blunt ends and cloned into the *Bam*HI/*Sal*I sites of *luxCDABE* fusion vector pFU175. Plasmids pFU194–198 were constructed to analyse the effect of promoter P*_invA_*_O:3_ truncations. This was accomplished by separate cloning of five DNA fragments amplified with primers II542–546 and II178 from genomic DNA of YeO:3 Y11 into the *Bam*HI/*Sal*I sites of pFU175. Plasmids pFU201 and pFU202 carry the *invA*O:3 promoter region from position −248 to −146 and from −448 to −146, respectively, fused to the *luxCDABE* operon. For their construction PCR fragments were amplified with primer pairs II570/II543 and II571/II543, respectively, and integrated into the *Bam*HI/*Sal*I sites of pFU175. The P*_rovA_*::*lacZ* fusions plasmids pFU129 and pFU130 were constructed by insertion of *rovA* promoter fragments, amplified with primer pair II260/I277 from genomic DNA of YeO:8 strain 8081v or YeO:3 strain Y11, into the *Bam*HI/*Sal*I sites of pFU109.

Following plasmids were engineered for the construction of *rovA*, *invA* and *yadA* mutant strains of *Y. enterocolitica*. For *rovA* mutagenesis, plasmid pFU167 was constructed by amplification of *sacB* with primers II421/II422 from pJE4 and integration into the *Avr*II/*Not*I sites of pFU102. pFU102 was constructed by insertion of three PCR-derived fragments into the *Spe*I/*Not*I sites of pFU100: (i) a *Spe*I/*Sac*I fragment containing 179 bp of the *rovA* regulatory region amplified with primers II171/II172, (ii) a *Sac*I/*Aat*II fragment encoding the chloramphenicol resistance gene of pZA31 luc, and (iii) a *Aat*II/*Not*I containing 119 bp of the downstream region of *rovA* amplified with primers II173/II174. For the *invA* mutagenesis, plasmid pFU213 was constructed by insertion of the *Sac*I/*Aat*II fragment encoding the kanamycin resistance gene of pZS*24MCS into pFU114. The plasmid pFU114 was constructed by insertion of an ‘*invA*’ fragment into the *Sal*I/*Not*I sites of pFU100. The PCR fragment contained base pairs 40–339 of the *invA* coding region, amplified from genomic DNA of YeO:3 Y11 with primer pairs II211/II212 which introduce stop codons at the 5′ and 3′ end in the open reading frame of the ‘*invA*’ fragment. For *yadA* mutagenesis, plasmid pFU187 was used. This plasmid was engineered by the insertion of three PCR fragments into the *Spe*I/*Not*I sites of pFU167: (i) a *Spe*I/*Sac*I fragment which contained 150 bp of the *yadA* regulatory region amplified from genomic DNA of YeO:3 Y11 with primer pair II513/II514 (ii) a *Sac*I/*Aat*II fragment of pFU32 encoding the tetracycline resistance gene, (iii) and a *Aat*II/*Not*I fragment containing the last 81 bp of the *yadA* gene plus 69 bp of the *yadA* downstream region amplified from genomic DNA of YeO:3 Y11 with primer pair II515/II516.

For deletion of the IS1667 element in the *invA*O:3 regulatory region, we used pFU190. Plasmid pFU190 was created by the insertion of three fragments. Two fragments starting from position −1887 to −1627 and −249 to −1 of the *invA* promoter region were amplified with primer pairs II519/II522 and II523/II524, respectively, and ligated at their blunt ends. The resulting *Xho*I/*Not*I fragment was ligated with the *Spe*I/*Xho*I fragment from pFU32 harboring the tetracycline resistance cassette and was inserted into the *Spe*I/*Not*I sites of pFU167.

Plasmids pFU184 and pFU185 were used for chromosomal integration of a *rovA* wild-type and a *rovA*_S98P_ mutant copy. Both plasmids were constructed by insertion of the *Sac*I/*Avr*II fragment harboring the R6K ori and the mobRP4 mobilization region from pFU100 into pFU119 and pFU138, respectively. For the generation of pFU119 *rovA*_O:3_ including its regulatory region was amplificated from genomic DNA of YeO:3 strain Y11 with primer pair II260/II226 and cloned into the *BamH*I/*Not*I sites of pFU109. pFU138 was obtained by QuikChange mutagenesis (Stratagene) of pFU119 using primer II377/II378. All clones were confirmed by sequencing (GATC, Konstanz, Germany).

### Construction *Y. enterocolitica* mutant strains

To construct the *rovA*, *invA*, and *yadA* mutants, the suicide plasmids carrying an internal fragment of *invA* with integrated stop codons (pFU213), or the insertion mutations *rovA*::Cm^R^ (pFU167) or *yadA*::Tet^R^ (pFU187) were propagated in *E. coli* S17-1 λpir and introduced by mobilization into YeO:3 strain Y1 and Y11. Transconjugants were selected on *Yersinia* selective agar (Oxoid) supplemented with antibiotics, selecting for the resistance of the plasmids. Since the suicide vectors cannot replicate in the YeO:3 strains, the obtained colonies are the result of plasmid integration into the *Yersinia* chromosome at regions of homology. The recombination event yielded merodiploid strains, which includes a wild-type and a mutant copy. Spontaneous second site recombinants where the integrated suicide vector and the wild-type copy of the gene were eliminated were isolated by selecting fast growing transconjugants on 10% sucrose plates. The *rovA* gene of YeO:3 (*rovA*_O:3_) and YeO:8 (*rovA*_O:8_) were introduced into the Δ*rovA* strain YE12 by conjugation. Loss of the gene function in resulting mutant strains YE1 (Δ*rovA*), YE12 (Δ*rovA*), YE18 (Δ*yadA*) and YE21 (Δ*invA*) and regain of RovA production in strains YE13 (*rovA*_O:3_) and YE14 (*rovA*_O:8_) was verified by PCR and western blotting analysis. Deletion of the IS1667 element in P*_invA_*_O:3_ was achieved by conjugation of plasmid pFU190 into YeO:3 strain Y1 as described above. All deletions and reintegration were verified by PCR.

### Quantitative real-time PCR

Quantitative RT-PCR was performed in triplicate with independent RNA preparations using a Rotor-Gene Q thermo cycler (Qiagen). RNA was prepared using the RNeasy Mini Kit (Qiagen) according to the manufacturer's protocol. 1 µg total RNA was taken for cDNA synthesis using the QuantiTect Reverse Transcription Kit (Qiagen) according to the manufacturer's instructions. For quantitative RT-PCR, reagents from Qiagen QuantiTect SYBR Green PCR KIT (Qiagen) were used. Gene specific-primers used for qRT-PCR amplification are listed in Table **S2** and were designed to produce a 120–150 bp amplicon. The amount of PCR product was quantified by measuring fluorescence of SYBR Green dye. Reported gene expression levels were normalized to levels of the 5S rRNA. Standard curves were detected during every run for each gene tested and established by comparing transcript levels in serial dilutions of total RNA from a control sample.

### Primer extension analysis to determine the transcriptional start sites of the *invA* and the IS1667-specific promoter

Primer extension analysis was performed to determine the transcription start points of the *invA* gene in strain Y1. *Y. enterocolitica* Y1 was grown in LB at 25°C to an OD_600_ of 3.0 (stationary phase). Total RNA was extracted of the samples using the SV total RNA purification kit (Promega) as described by the manufacturer. Annealing was performed with 20 µg extracted RNA and the 5′-Dig-labelled oligonucleotides (primer III94 for the IS1667 specific promoter, primer III91 for the *invA* promoter) in 20 µl of 1× First Strand Buffer (Invitrogen) by slow cooling of the sample (0.01°C/sec) including 8 mM dNTPs and 5× FS Buffer (Invitrogen). 200 U Superscript II reverse transcriptase (Invitrogen) was added and incubated for 1 h at 42°C. The size of the Dig-labelled reaction products was determined on a denaturing 4% DNA sequencing gel by a detection procedure as described [68].

### Luciferase and beta-galactosidase assays

Optical density (OD_600_) of three independent cultures of the bacteria harboring the different luciferase reporter plasmids was determined and diluted to an OD_600_ of 0.1 to monitor growth of the bacteria at indicated growth conditions (complex media, 25°C and 37°C). In parallel, bioluminescence was detected in non-permeabilized cells with a Varioskan Flash (Thermo Scientific) using the SkanIt software (Thermo Scientific). Bioluminescence was measured for 1 s every 30 min and is given as relative light units (RLU/OD_600_) from three independent cultures performed in duplicate. Beta-galactosidase activity was determined as described [44]. The activities were calculated as follows: beta-galactosidase activity OD_420_ • 6.75 OD_600_^−1^ • Δt (min)^−1^ • vol (ml)^−1^.

### Purification of *Y. enterocolitica* RovA and H-NS

RovA proteins were overexpressed in *E. coli* BL21 CodonPlus (DE3)-RIL, H-NS was expressed in *E. coli* KB4. Overnight cultures of *E. coli* strains, harbouring the plasmids pFU156 (*rovA*_O:8_-*his*_6_), pFU157 (*rovA*_O:3_-*his*_6_) or pFU199 (*his*_6_-*hns*) were diluted 1∶100 and grown at 37°C in LB medium for 2 h. Subsequently, protein synthesis was induced with 100 µM IPTG (pFU156, pFU157) or 200 µg/l AHT (pFU199) and grown for 4 h. Bacteria overexpressing His-tagged RovA proteins were purified as described [55].

### Gel electrophoresis, preparation of cell extracts and western blotting

For the analysis of *invA* and *rovA* expression bacteria were grown under environmental conditions as described. The optical density of the cultures was adjusted and a 1 ml aliquot was withdrawn from each culture. The cells were collected by centrifugation, and resuspended in 100 µl sample buffer (100 mM Tris-HCl pH 6,8, 2% SDS, 10% glycerol, 3% DTT, 0.001% bromophenol blue) and analyzed by gel electrophoresis and western blotting. For the immunological detection of the InvA, YadA and RovA proteins, the cell extracts were separated on 10% or 15% SDS-polyacrylamide gels, and the proteins were separated by electrophoresis and transferred onto an Immobilon membrane (Millipore). Identity and expression of the adhesins were confirmed by westernblotting analysis using polyclonal antibodies against the *Y. enterocolitica* invasin or the *Y. pseudotuberculosis* RovA and YadA protein, and a second goat alkaline-phosphate antibody (Sigma) using 5-bromo-4-chloro-3-indoylphosphate (XP) and nitroblue tetrazolium (Boehringer Mannheim) as substrates.

### *In vivo* stability analysis of RovA

Protein biosynthesis of bacterial cultures in exponential phase at 37°C was stopped by adding 50 µg/ml gentamycin and 50 µg/ml tetracycline. Samples were taken at indicated time points.

### Gel retardation assays

Binding of RovA to defined PCR fragments carrying different portions of the *invA* regulatory region was carried out in a 20 µl reaction mixture containing increasing amounts of purified RovA protein (0.5–1.5 µg) and 80 ng of DNA. The reaction buffer contained 10 mM Tris-HCl, pH 7.5, 1 mM EDTA, 50 mM NaCl, 5 mM MgCl_2_, 5 mM dithiothreitol (DTT) and 5% glycerol. The reaction mixture was incubated for 20 min at room temperature or at 37°C and separated on polyacrylamide gels as described [44]. PCR fragments encoding the *invA* promoter fragments a, b and c were amplified with primer pairs II519/II551, II546/II178 and II558/II559, and the *csiD* promoter fragment was produced by PCR with primer pairs 131/132.

### Motility assays

A 2 µl aliquot of an overnight culture grown at 25°C in LB medium was spotted onto semisolid agar plates containing 0.35% agar to evaluate motility [69]. The capacity of YeO:3 Y1 and YeO:8 8081v to spread was monitored after 48 h at 25°C and 37°C.

### Electron microscopy

YeO3 Y1 and YeO8 8081v grown at 25°C and 37°C overnight were fixed in growth medium with 1% formaldehyde. For transmission electron microscopy thin carbon support films were prepared by sublimation of carbon on freshly cleaved mica. Using 300 mesh copper grids, the samples were negatively stained with 2% (w/v) aqueous uranylacetate, according to the method of [70], and examined in a transmission electron microscope (TEM910, Zeiss, Germany) at an acceleration voltage of 80 kV at calibrated magnifications. Images were recorded digitally with a Slow-Scan CCD camera (ProScan 1024×1024, Scheuring, Germany) with ITEM software (Olympus Soft Imaging Solutions, Münster, Germany). Images were corrected for brightness and contrast applying Adobe photoshop CS3.

For field emission scanning electron microscopy glass coverslips samples were fixed with a solution containing 5% formaldehyde and 2% glutaraldyhde in cacodylate buffer (0.1 M cacodylate, 0.01 M CaCl_2_, 0.01 M MgCl_2_, 0.09 M sucrose pH 6.9). Dehydration was carried out in a graded series of acetone (10%, 30%, 50%, 70%, 90%, 100%) on ice for 15 min for each step. Samples were then critical-point dried with liquid CO_2_ (CPD 030, Balzers Union, Liechtenstein) and covered with a gold film by sputter coating (SCD 040, Balzers Union, Liechtenstein). For examination in a field emission scanning electron microscope (DSM 982 Gemini, Zeiss, Germany), an Everhart Thornley SE detector was used with the inlens SE detector in a 50∶50 ratio at an acceleration voltage of 5 kV.

### Cell adhesion and invasion assay

For cell adhesion and uptake assay 5×10^4^ HEp-2 cells were seeded and grown overnight in individual wells of 24-well cell culture plates. Cell monolayers were washed three times with PBS and incubated in binding buffer (RPMI 1640 medium supplemented with 20 mM HEPES (pH 7.0) and 0.4% BSA before the addition of bacteria. Approximately 5×10^5^ bacteria were added to the monolayer and incubated without or after centrifugation of the bacteria onto the monolayer at 22–25°C to prevent bacterial internalization and 37°C to test for cell binding and invasion as described [62], [71]. 30 min post infection, the cells were washed extensively with PBS. The total number of host cell-associated bacteria was determined by cell lysis using 0.1% Triton X-100 and plating on bacterial media. Bacterial uptake was assessed 30 min after infection as the percentage of bacteria, which survived killing by gentamicin, as described [63]. For each strain, the relative level of bacterial adhesion and uptake was determined by calculating the number of colony-forming units relative to the total number of bacteria introduced onto monolayers. Number of invaded bacteria is given relative to the number of cell-bound bacteria. The experiments were routinely performed in triplicate.

### Mouse infection

Bacteria used for oral infection were grown overnight in LB medium at 25°C, washed and resuspended in PBS. Female BALB/c mice 6–8 weeks old were purchased by Janvier. Groups of 7–10 animals were pretreated with desferal 24 h prior infection as described previously [72]. Subsequently, mice were orally infected with *Y. enterocolitica* strains Y1, YE15 or YE14 in single infection and co-infection experiments using a ball-tipped feeding needle. 5×10^8^ bacteria of each strain were administered orogastrically. In co-infection experiments, mice were orally infected with an equal mixture of 5×10^7^ (low dose) or 5×10^8^ (high dose) bacteria of *Y. enterocolitica* strains Y1 and YE14, or Y1 and YE15. For discrimination of strains Y1 and YE15 low-copy vectors pFU99 and pFU109 with different antibiotic resistance cassettes were introduced in Y1 and Y15, respectively. Presence of these vectors had no effect on *Yersinia* fitness and virulence and they were maintained in all bacteria recovered from host tissues throughout a five days time course of infection (F. Uliczka, unpublished results). Three or five days after infection, mice were euthanized by CO_2_. Peyer's patches, mesenteric lymph nodes, liver and spleen were isolated. The ileum was rinsed with sterile PBS and incubated with 100 µg/ml gentamicin in order to kill bacteria on the luminal surface. After 30 min, gentamicin was removed by extensive washing with PBS for three times. Subsequently, all organs were weighed and homogenized in sterile PBS at 30.000 rpm for 30 sec using a Polytron PT 2100 homogenizer (Kinematica, Switzerland). The numbers of bacteria were determined by plating three independent serial dilutions of the homogenates on LB plates with and without antibiotics. The colony forming units (cfu) were counted and are given as cfu per g organ/tissue. The competitive index relative to wild-type strain Y1 was calculated as described by Monk *et al.* 2008 [78].

## Supporting Information

Figure S1 ***Y. enterocolitica*** **O:3 interaction with epithelial cells.** Ten different *Y. enterocolitica* serotype O:3 isolates from human patients or pigs, *Y. enterocolitica* O:8 strain 8081v, *Y. enterocolitica* O:9 strain 4620 and *Y. enterocolitica* O:5,27 strain 3056 were grown at 25°C overnight in LB medium. About 5·10^4^ HEp-2 cells were infected with 5·10^5^ bacteria and incubated at 37°C to determine cell association (adhesion+invasion) and the internalization efficiency of the bacteria. *E. coli* K-12 was used as negative control. Data are presented as means ± standard deviations of three independent experiments performed in duplicate.
(EPS)

Figure S2 **Motility of YeO:3 within the intestinal tract of infected Balb/c mice.** Transmission electron microscopy of *Y. enterocolitica* O:3 strain Y1 isolated from the intestine of Balb/c mice three days after infection.
(EPS)

Figure S3 **Quantitative RT-PCR of** ***invA*** **expression.** Quantitative RT-PCR was performed on total RNA extracted from the *Y. enterocolitica* wild-type strain Y1, the isogenic *rovA* deletion mutant (YE12), the reconstituted *rovA* ^+^ strain (YE13), and the IS1667 (YE15) deletion mutant grown at 37°C to stationary growth phase. All values are expressed as relative mRNA levels compared to expression levels of the wild-type Y1. The calculated ratios in the relative *invA* expression levels between wild-type and the *rovA* or the IS1667 deletion mutant determined by quantitative RT-PCR and *invA-lux* fusions are given below the graph.
(EPS)

Figure S4 **Mapping of the transcriptional start sites by primer extension analysis.** Twenty µg of total RNA isolated of *Y. enterocolitica* O:3 strain Y1 grown at 25°C were used as template RNA with primers specific for the *invA* regulatory region. The sequencing ladders are shown on the left. The arrows mark the +1 transcriptional start sites within the IS1667 element (**A**) and the *invA* promoter region (**B**). The putative −10 region is given in bold letters.
(EPS)

Figure S5 **Interaction of RovA_O:3(S98)_ and RovA_O:8(P98)_ with the** ***invA*** **regulatory region at 25°C and 37°C.** (**A**) Overview of the *invA* promoter region of *Y. enterocolitica* O:3 strains. The transcriptional start sites of the *invA* promoter and of the predicted IS1667-encoded promoter are indicated by broken arrows. The dark boxes represent the RovA and the white small boxes the H-NS binding sites identified in the homologous *invA* promoter of *Y. pseudotuberculosis* [46]. The thick line represents the *invA* promoter sequence and the thin line illustrates the sequence of the IS1667 element with the putative transposase gene. The fragment used for the band shift experiments is shown by a black line. (**B**) The double-stranded promoter fragment of the *invA* regulatory region harbouring one RovA-binding site was incubated without or with increasing concentrations of purified RovA_O:3(S98)_ or RovA_O:8(P98)_ at 25°C and 37°C. The DNA-protein complexes were separated on a 4% polyacrylamide gel. A non-specific probe containing weight standard 100 bp ladder was loaded on the left. The higher molecular weight protein-DNA complexes and the positions of the non-shifted control fragments are indicated.
(EPS)

Figure S6 **Analysis of RovA expression in** ***Y. enterocolitica*** **O:3.** *Y. enterocolitica* strains YeO:8 8081v, YeO3, Y11 and an isogenic *rovA* mutant of Y11 (YE01) harboring the fusion plasmid P*_rovA_*_O:8_::*lacZ* or P*_rovA_*_O:3_::*lacZ* were grown at 25°C and 37°C overnight. The beta-galactosidase activity determined from the cultures is given in µmol min^−1^ mg^−1^ and represents the mean ± standard deviation of at least three independent experiments.
(EPS)

Table S1 ***Y. enterocolitica*** **O:3 isolates.** * date of first citation found in literature/isolation before indicated time point. ^#^Analysis of the recently sequenced genomes of twenty *Y. enterocolitica* O:3 strains isolated in Great Britain between 1999 and 2002 revealed that all of them contained an IS1667 insertion at position −143 with respect to the start codon of the *invA* gene and the *rovA*_S98_ allele (Alan McNally, personal communication).
(DOC)

Table S2 **Primers.** Restriction sites used for cloning procedures are underlined.
(DOC)
