# Supplementary material for: Unique Cell Adhesion and Invasion Properties of Yersinia enterocolitica O:3, the Most Frequent Cause of Human Yersiniosis
Source: PLoS Pathog. 2011 Jul 7;7(7):e1002117. doi: 10.1371/journal.ppat.1002117 (PMC3131269; doi:10.1371/journal.ppat.1002117)
Supplement: Table S1 — Y. enterocolitica O:3 isolates. * date of first citation found in literature/isolation before indicated time point. #Analysis of the recently sequenced genomes of twenty Y. enterocolitica O:3 strains isolated in Great Britain between 1999 and 2002 revealed that all of them contained an IS1667 insertion at position −143 with respect to the start codon of the invA gene and the rovA S98 allele (Alan McNally, personal communication). (DOC) [file ppat.1002117.s007.doc]

**Table S1:** *Y. enterocolitica* O:3 isolates.

**Isolate Serotype Biotype Origin Country Year P*_invA_* IS1667 *rovA* allele**

Y1 O:3 4 human Germany 2007 yes *rovA*_S98_

Y2 O:3 4 human Germany 2007 yes *rovA*_S98_

Y3 O:3 4 human Germany 2007 yes *rovA*_S98_

Y4 O:3 4 human Germany 2007 yes *rovA*_S98_

Y5 O:3 4 human Germany 2007 yes *rovA*_S98_

Y8 O:3 4 human Germany 2007 yes *rovA*_S98_

Y9 O:3 4 human Germany 2007 yes *rovA*_S98_

Y10 O:3 4 human Germany 2007 yes *rovA*_S98_

Y11s O:3 4 human Germany 1994 yes *rovA*_S98_

05-06546 O:3 4 human Germany 2006 yes *rovA*_S98_

05-06716 O:3 4 human Germany 2005 yes *rovA*_S98_

05-06794 O:3 4 human Germany 2006 yes *rovA*_S98_

05-06795 O:3 4 human Germany 2006 yes *rovA*_S98_

06-03446 O:3 4 human Germany 2006 yes *rovA*_S98_

06-05025 O:3 4 human Germany 2006 yes *rovA*_S98_

08-01985 O:3 4 human Germany 2008 yes *rovA*_S98_

JH 5700/84 O:3 4 human Germany 1984 yes *rovA*_S98_

JH 1131/84 O:3 4 human Germany 1984 yes *rovA*_S98_

Y32 O:3 4 porcine Germany 2007 yes *rovA*_S98_

Y33 O:3 4 porcine Germany 2007 yes *rovA*_S98_

Y34 O:3 4 porcine Germany 2007 yes *rovA*_S98_

Y35 O:3 4 porcine Germany 2007 yes *rovA*_S98_

Y37 O:3 4 porcine Germany 2007 yes *rovA*_S98_

Y38 O:3 4 porcine Germany 2007 yes *rovA*_S98_

Y39 O:3 4 porcine Germany 2007 yes *rovA*_S98_

Y40 O:3 4 porcine Germany 2007 yes *rovA*_S98_

Y41 O:3 4 porcine Germany 2007 yes *rovA*_S98_

Y22 O:3 4 porcine Germany 2007 yes *rovA*_S98_

PL-6 O:3 4 ? Poland ? yes *rovA*_S98_

PL-15 O:3 4 human Poland ? yes *rovA*_S98_

PL-20 O:3 4 ? Poland ? yes *rovA*_S98_

YeO3 O:3 4 human Finland 1976 yes *rovA*_S98_

80016 O:3 4 human Finland 2006 yes *rovA*_S98_

84053 O:3 4 human Finland 2006 yes *rovA*_S98_

37 O:3 4 human Finland 1985 yes *rovA*_S98_

1870/73 O:3 4 ? Finland 1973 yes *rovA*_S98_

1150/73 O:3 4 ? Finland 1973 yes *rovA*_S98_

C34 M2 O:3 4 ? Europe 1983* yes *rovA*_S98_

RB C36 M3 O:3 4 ? Europe 1983* yes *rovA*_S98_

C32 M1 O:3 4 ? Europe 1983* yes *rovA*_S98_

Y748 O:3 4 ? France ? yes *rovA*_S98_

Y751 O:3 4 ? Great Britain^#^ ? yes *rovA*_S98_

Y756 O:3 4 ? South Africa ? yes *rovA*_S98_

Y752 O:3 4 ? Brasil ? yes *rovA*_S98_

Y765 O:3 4 ? Australia ? yes *rovA*_P98_

Y745 O:3 4 ? Japan ? yes *rovA*_P98_

Y746 O:3 4 ? Canada ? yes *rovA*_P98_

E675 O:3 4 ? North America 1983* yes *rovA*_P98_

JD E675 O:3 4 ? North America 1985* yes *rovA*_P98_
