# Supplementary material for: Unique Cell Adhesion and Invasion Properties of Yersinia enterocolitica O:3, the Most Frequent Cause of Human Yersiniosis
Source: PLoS Pathog. 2011 Jul 7;7(7):e1002117. doi: 10.1371/journal.ppat.1002117 (PMC3131269; doi:10.1371/journal.ppat.1002117)
Supplement: Table S2 — Primers. Restriction sites used for cloning procedures are underlined. (DOC) [file ppat.1002117.s008.doc]

**Table S2:** Primers

Primer Description

131 GTC CTG GCC TGA ATC GAC AGC G

132 GG CTA CGA AAT GAG CAT CGC

II40 GCA CGG AGC TCA GGA GGA ATT AAC C ATG TAT TCA TTT TTT AAT

ACG CTA AC

II42 GCA CAG TCG ACC TAT TGA GGC TCC GCA CAC

II171 GCA CGA CTA GTA GGT TAA TCC TTA AGA GGT AAT T

II172 GCA CGG AGC TCT TGC CCC TCC TTT TAT TTA G

II173 GCA CGG ACG TCA ATA AAG TTT TAA GAC TCA ATT CAC

II174 GCA CAG CGG CCG CCG CCT CTC TGT AAT ATA TTT TAT GA

II177 GCA CGG GAT CCT CCA ATA TGG ATA AGT GAA GG

II178 GCA CGG TCG ACT AGT GTA CCC CTT AGG TAT AAA TTA AC

II211 GCA CTG TCG ACT GAA TTA GCA GGC TAA TAT TAT CGA TC

II212 GCA CTG CGG CCG CTT AAT CAA GAG AAC TTT CTT TGA GG

II226 GCA CTG CGG CCG CCG CCT CTC TGT AAT ATA TTT TAT GA

II260 GCA GTG GAT CCG CCA GCG AAA TGG TGC AGG T

II277 GCA CGG TCG ACT TAC AGA TCC TAA TGT CGA TTC CA

II375 GAC GGA ACA GTC TTC ATC GAT AAT AGA GCA GGT

II376 ACC TGC TCT ATT ATC GAT GAA GAC TGT TCC GTC

II377 GAC GGA ACA GTC TTC ACC GAT AAT AGA GCA GGT

II378 ACC TGC TCT ATT ATC GGT GAA GAC TGT TCC GTC

II417 GCA CGC CAT GGA ATC GAC ATT AGG ATC TG

II418 GCA CGC TCG AGC TTA CTT TGT AGT TGA ATA ATG TTT C

II421 GCA CGC CTA GGG ATC CTT TTT AAC CCA TCA C

II422 GCA CAG CGG CCG CAA AAG GTT AGG AAT ACG GTT AG

II513 GCA CGA CTA GTT GCT GTT TTT TGC ATG ATT ATC

II514 GCA CGG AGC TCT GTA AGC ACC TTT TAT ATT TCT TT

II515 GCA CGG ACG TCA ATG TCG CAC TTA AAG CCG

II516 GCA CAG CGG CCG CAT GAA TAA AAC ACC GAT TAC ATA ATC

II517 GCA CGC CAT GGT GAC TAA AGA TTT TAA GAT CAG TGT C

II518 GCA CGG TCG ACT TAC CAT TCG ATA TTA AAT GAT GC

II519 GCA CGC TCG AGA TCC AAT ATG GAT AAG TGA AGG C

II522 TGA TAT GTT ATT ATT ATC AAA ATA GC

II523 TAT ATA TAT TCA GGC TAA ATA TAA CCT G

II524 GCA CGG CGG CCG CTA GTG TAC CCC TTA GGT ATA AAT TAA C

II542 GCA CTG GAT CC GGC CTG TGC CAC AAC GGC

II543 GCA CAG GAT CC GGG GGA ATG AGTTTA ACC TC

II544 GCA CTG GAT CC GTC GAT ACC AAG AGT ATA TAC TTT CATG

II545 GCA CTG GAT CC CAT TAT ATATAT TCA GGCTAA ATA TAA CCT

II546 GCA CTG GAT CC TGC ATT TCA TTT GTT ATT GCT G

II551 GGT TTA TTG GGA GCG TCC BS

II558 GAA AAG GGA GGA ATT TAT CGC G

II559 GAA ATG GAC GCT CCA ACT TTT AC

II570 GCA CGG TCG ACT TGG CAA AAA ATG TTT TCC AG

II571 GCA CGG TCG ACA TGA AAT GGA CGC TCC AAC T

II726 GCA CGG AAT TCA GCG AAG CGT TAA AGA TTC TTA AC

II727 GCA CGG TCG ACT TAC AGC AGG AAA TCA TCC AG

II812 GCC TGG CGG CCA TAG CGC (5S rRNA, qRT-PCR)

II813 GCC TGG CAG TGT CCT ACT CT (5S rRNA, qRT-PCR)

III95 ACC CCG CTG AAC ATA ATG AG (*invA*, qRT-PCR)

III96 TGC CGC GTC ATT TAC CAT TG (*invA*, qRT-PCR)

III91 DIG-TAG TGT ACC CCT TAG GTA TAA ATT AAC (primer extension for P*_invA_*)

III94 DIG-TCA TCA TGG TAA TAT TAG CTT GCT (primer extension for P_IS1667_)
